# Emotional demands and burnout: Differential moderating effect of job resources and age

**DOI:** 10.1371/journal.pone.0351940

**Published:** 2026-06-22

**Authors:** Tímea Zsuzsanna Popucza

**Affiliations:** Department of Occupational Health, Psychology and Sports Science, Faculty of Health and Occupational Studies, University of Gävle, Gävle, Sweden; Nanjing University, CHINA

## Abstract

This study, based on the Job Demands–Resources (JD-R) model, investigates how perceived and contact-related emotional demands contribute to burnout, and whether job resources -deep acting, meaning of work, work control, and social support at work-buffer these effects. A cross-sectional online survey was conducted among professionals in healthcare, care, and human service roles (*N =* 1,506). Structural Equation Modelling (PLS-SEM) was used to examine moderating effects of job resources on the relationships between emotional demands and the burnout dimensions of exhaustion and disengagement. Both types of emotional demands were positively associated with both burnout dimensions. While job resources were generally negatively associated with exhaustion and disengagement, they did not moderate the effects of emotional demands in the expected buffering direction. Instead, meaning of work and social support amplified the relationships between contact-related and perceived emotional demands, respectively, and exhaustion. Exploratory analyses revealed no significant linear or curvilinear age-based moderation, suggesting that these moderation patterns function similarly across age groups. Overall, the findings indicate that while job resources may reduce general burnout, they are not universally protective in emotionally demanding work. This study highlights the need to refine the JD-R assumption that resources consistently buffer job demands and underscores the importance of identifying alternative resources and targeted interventions in emotionally demanding roles.

## Introduction

Burnout has long been recognized as a major occupational health hazard, particularly in professions involving interpersonal interaction and emotional involvement, such as healthcare, education, and social work [1–2]. While originally conceptualized within human service roles, burnout is now widely acknowledged as a broader workplace phenomenon affecting a range of sectors, including customer service, retail, care and healthcare work, and other relational occupations. In today’s increasingly service-oriented labour markets, burnout has become a pressing public health concern. Although it originates in the workplace, its consequences often extend beyond it, contributing to negative health outcomes such as depression, anxiety, insomnia, and reduced quality of life [3]. These wide-reaching effects underscore the importance of understanding not only what drives burnout but also what resources can help prevent it and promote long-term occupational health and sustainability.

In this study, burnout is conceptualized within the Job Demands–Resources (JD-R) model, which defines it as a strain response resulting from an imbalance between high job demands and insufficient resources [4]. More specifically, burnout is operationalized through two core dimensions of exhaustion and disengagement. Exhaustion reflects the depletion of emotional, physical, and cognitive energy due to prolonged exposure to demanding work conditions, and disengagement describes emotional, cognitive, and behavioural withdrawal from work and growing negativity toward tasks and the work environment [5–6]. Among the various job demands that contribute to burnout, emotional demands have received increasing attention, particularly in occupations where interpersonal contact is a central part of the daily work.

Within the Job Demands–Resources (JD–R) model, emotional demands refer to job aspects that require emotional effort and, when not adequately counterbalanced by resources, can lead to strain and negative outcomes such as exhaustion and burnout [4,7]. They are often defined as emotionally charged interactions that workers encounter as part of their job, such as managing difficult client interactions or facing distressing situations, which require self-control and emotion regulation in the workplace [8–9]. In contrast to emotional labour, which focuses on the strategies employees use to regulate emotions in line with organizational display rules, such as surface and deep acting [10–11], emotional demands are understood as a broader job characteristic. They encompass both externally imposed interpersonal challenges and the internal psychological strain they provoke [4,7]. Over time, emotional demands have been conceptualized in different ways: some approaches focus on exposure to emotionally challenging job content, for example, encounters with pain, death, difficult interactions, and professional worries [12–14], whereas others emphasize employees’ subjective experiences of emotionally taxing work, as in broader psychosocial frameworks [15–18]. Thus, emotional demands can be assessed in both objective and subjective terms: through indicators of job content, such as the frequency and intensity of exposure to difficult or distressing interactions, and through employees’ appraisals of how emotionally demanding their work is [9]. This dual perspective captures both the structural characteristics of work and the lived psychological strain they generate. However, some conceptualizations conflate emotional demands with employees’ efforts to regulate or display emotions (e.g., hiding feelings), which risks blurring the distinction between job-related emotional exposures and the emotion regulatory processes individuals use to manage them. In this study, we conceptualize emotional demands as including both exposure to difficult or distressing interactions and the subjective experience of how emotionally demanding work is [9], while distinguishing these demands from regulatory strategies such as deep acting.

While emotional demands represent a central type of job demand within the JD–R model, employees’ well-being is also shaped by the resources available to them. Job resources are aspects of the work environment or personal characteristics that help achieve work goals, promote personal development, and mitigate the negative effects of high job demands on employee strain [4,7]. Resources can be organizational (provided by the workplace) or personal (the workers’ capacity). Organizational resources include workplace-related factors such as job autonomy (i.e., decision latitude) and social support at work, which enhance employees’ control over their work situation and provide both practical and emotional support from colleagues and supervisors. Personal resources, on the other hand, are internal psychological traits or capacities, such as self-efficacy, optimism, and the perceived meaningfulness of one´s work, that promote resilience and help individuals cope more effectively with stressors [19,20]. Although the JD–R model posits that job resources can buffer the negative effects of job demands, the extent to which such buffering applies to emotionally demanding work remains uncertain. In particular, it is uncertain whether different personal and organizational resources uniformly attenuate the impact of emotional demands on burnout.

In the present study, we consider workers’ ability to use deep acting as a personal resource. Deep acting, a form of emotional labour, involves aligning one’s genuine emotional state with externally expected expressions [10,11]. Unlike surface acting, which refers to the suppression or faking of emotions and has been consistently associated with strain, emotional dissonance, and burnout [21], deep acting reflects a more constructive emotion regulatory strategy linked to positive outcomes such as personal accomplishment, positive affect, job satisfaction, and well-being (see Hülsheger and Schewe [21] for a review). Evidence further suggests that surface acting functions as a strain mechanism, mediating the relationship between emotional demands and burnout [22–25]. By contrast, deep acting has neither shown consistent associations with negative health outcomes [21] nor been found to mediate the relationship between emotional demands and strain outcomes [22–24]. Instead, it may be better understood as a personal resource that helps mitigate the negative effects of emotional demands on health. Conceptually, deep acting also overlaps with cognitive reappraisal, an antecedent-focused emotion regulation strategy that facilitates positive emotional experiences and psychological well-being [11,26]. Taken together, the empirical findings, the overlap with cognitive reappraisal, and the potential protective function position deep acting as a personal resource within the JD–R model.

In addition to resources, age may also play a role in how employees experience and respond to emotional demands in the workplace. Several reviews and meta-analyses suggest that age may be associated with better mental health and lower burnout in occupational settings, though findings are inconsistent. A meta-analysis by Brewer and Shapard [27] found a small but consistent negative correlation between age and emotional exhaustion, suggesting that older workers tend to report lower burnout. Similarly, systematic reviews by Baxter et al. [28] and Mori et al. [29] found that older employees often show neutral or favourable mental health outcomes, particularly when employed in supportive work environments that offer autonomy, low demands, and strong support. These findings suggest that the benefits of age are not universal but may be contingent on the quality of the psychosocial work environment.

The Socioemotional Selectivity Theory (SST) proposes that older adults experience increased emotional well-being because they prioritize emotionally meaningful goals and social interactions as their perceived time horizons shorten [30]. In the work environment, this motivational shift may be linked to enhanced emotion regulation capacities in later adulthood [31]. For instance, older employees are more likely to engage in deep acting [31], which may support well-being and buffer against burnout in later life. However, according to the Strength and Vulnerability Integration (SAVI) model [32], when emotional stressors are intense, age-related declines in physiological flexibility may leave older adults more vulnerable to strain. Moreover, the SWEAGE model [33] emphasizes that the cumulative impact of different job demands over time can erode older workers’ well-being, particularly in the absence of adequate resources. Furthermore, the observed improvements in emotional functioning with age may partly reflect a selection effect, commonly referred to as the Healthy Worker Effect, in which individuals who are less able to meet the emotional or physical demands of work are more likely to exit the workforce earlier [34]. Taken together, while aging may enhance emotion regulation and potentially increase one’s access to or reliance on organizational and personal resources, how these age-related differences interact with resources that may protect against the negative effects of high emotional demands on burnout remains poorly understood.

As the workforce continues to age and the retention and well-being of older employees become increasingly important organizational priorities, there remains limited empirical evidence on whether the moderating effects of job resources on emotional demands vary across age groups. Given the competing possibilities, this study adopted an exploratory approach to assess whether age further moderates the buffering effects of personal resources (such as deep acting and meaning of work) and organizational resources (such as job control and social support at work) on the relationship between emotional demands and burnout. Accordingly, follow-up analyses of three-way interactions were conducted in cases where resources significantly moderated the direct effects in our main model. This approach allows us to explore whether and how age influences the protective function of work resources in emotionally demanding environments.

### Aim

**Fig 1:**
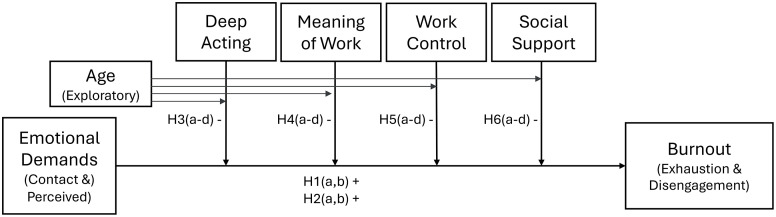
The principal model.

The primary aim of this study is to investigate the relationship between emotional demands and burnout, focusing on its two core dimensions: exhaustion and disengagement, within a broad Swedish occupational context that includes workers from healthcare, care, and other human-facing occupations beyond the care sector. The secondary aim is to examine the moderating role of workers’ personal resources, such as deep acting and meaning of work, and organizational resources, such as control and social support at work, in these relationships. The tertiary aim is to explore whether age further moderates these effects through three-way interactions, specifically, whether workers’ age influences the extent to which personal and organizational resources buffer the relationship between emotional demands and burnout. The principal model is presented in Fig 1.

### Hypotheses

Drawing on the Job Demands–Resources (JD–R) model, emotional demands are expected to increase burnout, whereas job resources are assumed to reduce burnout and buffer the negative effects of demands. Accordingly, we hypothesized positive associations between emotional demands and burnout, and the moderating effects of personal and organizational resources.

### Direct effects

H1a: Contact-related emotional demands are positively associated with disengagement.

H1b: Contact-related emotional demands are positively associated with exhaustion.

H2a: Perceived emotional demands are positively associated with disengagement.

H2b: Perceived emotional demands are positively associated with exhaustion.

### Moderation effects

H3a-d: Deep acting moderates the positive relationship between emotional demands (contact-related and perceived) and burnout (disengagement and exhaustion), such that higher levels of deep acting weaken these associations.

H4a-d: Meaning of work moderates the positive relationship between emotional demands (contact-related and perceived) and burnout (disengagement and exhaustion), such that higher levels of meaning weaken these associations.

H5a-d: Work control moderates the positive relationships between emotional demands (contact-related and perceived) and burnout (disengagement and exhaustion), such that higher levels of work control weaken these associations.

H6a-d: Social support moderates the positive relationships between emotional demands (contact-related and perceived) and burnout (disengagement and exhaustion), such that higher levels of social support weaken these associations.

Subscripts a–d denote moderation effects for (a) contact-related emotional demands and disengagement, (b) contact-related emotional demands and exhaustion, (c) perceived emotional demands and disengagement, and (d) perceived emotional demands and exhaustion.

## Methods

We conducted a cross-sectional online survey targeting Swedish-speaking professionals whose roles involve regular interaction with patients, care recipients, clients, or customers.

The study used data collected within the larger project Health and Emotional Burdens at Work. Ethical approval for the project, including the present study, was granted by the Swedish Ethical Review Authority (reference number Nr: 2023-08117-01). All procedures complied with the principles of the Declaration of Helsinki. The study is reported in accordance with the STROBE guidelines; the completed checklist is provided as Supplementary Material.

### Power analysis

A priori power analysis using G*Power 3.1 [35] was conducted to estimate the minimum required sample size for the hypothesized model. The analysis indicated that 822 responses were needed to detect small effects (f² = 0.02) at a power level of .80 and α = .05, assuming up to 10 predictors in the model, reflecting the inclusion of two types of emotional demands, four job resources, and four interaction terms. To ensure sufficient statistical power for both the main model and the additional exploratory three-way interaction analyses, which typically require larger samples to detect small effects, our goal was to recruit approximately 1,600 participants.

### Data collection

Participants were recruited through email invitations distributed via employers and targeted social media posts. Participation was voluntary, uncompensated, and conducted on a secure web platform. Informed consent was obtained electronically. Before accessing the survey, participants were presented with written information describing the study purpose, data handling procedures, their rights, and contact details for further information or concerns about participation. Participants indicated their consent by clicking either “Jag samtycker och vill börja att delta i studien” (“I consent and wish to participate in the study”) or “Jag samtycker inte och vill inte delta i studien” (“I do not consent and do not wish to participate in the study”). Only those who provided consent could proceed to the survey; those who declined were thanked for their interest and exited from the platform. No personal identifying information (e.g., name, email, or contact details) was collected, ensuring anonymous participation. Data collection took place in two stages. In the first phase (17 February–20 June 2024), employer-distributed email invitations yielded 33 responses. In the second phase (26 March–24 June 2024), social media posts shared via private accounts and in professional groups, as well as paid advertisements on social media platforms, generated 1,473 responses.

Eligibility criteria included: aged 18 and older, working in healthcare, care, or service roles, employed at ≥40% full-time (i.e., at least 16 hours per week), not on sick or personal leave for more than two weeks, and having regular direct interaction with patients, care recipients, clients, or customers. Participants self-selected into the occupational categories of healthcare (e.g., nurse, doctor, physiotherapist, psychologist), care (e.g., social worker, preschool teacher, eldercare worker), and other human service roles outside of care (e.g., teachers, librarians, receptionists, workers in retail, hospitality, and real estate, etc.). Participants were also encouraged to describe their roles in their own words.

In total, 1,506 respondents completed the survey. Of these, 1,406 (93.4%) identified as female, 93 (6.2%) as male, and 7 (0.5%) as unspecified. Ages ranged from 22 to 74 years (M = 46.40, SD = 10.39). A total of 17 missing values were automatically imputed using SmartPLS 4 [36]. Descriptive statistics, including occupational distributions, are reported in Table 1.

**Table 1:** Descriptive statistics, including occupational distributions.

Category	Total (N = 1,506)	Healthcare (n= 865)	Care (n = 453)	Service (n = 188)
Gender				
Female	1,406 (93.4%)	816 (94.3%)	425 (93.8%)	165 (87.8%)
Male	93 (6.2%)	47 (5.4%)	23 (5.1%)	23 (12.2%)
Unspecified	7 (0.5%)	2 (0.2%)	5 (1.1%)	0 (0.0%)
Age	M = 46.40 (10.39)	M = 45.70 (10.42)	M = 47.30 (10.34)	M = 47.53 (10.13)
Age range	22–74	22–69	22–72	24–74
Highest Education				
Elementary school or less	10 (0.7%)			
High school	153 (10.2%)			
<3 years post-secondary	213 (14.1%)			
3 years post-secondary	281 (18.7%)			
>3 years post-secondary	849 (56.4%)			
Work Experience				
<3 months	10 (0.7%)			
3–12 months	70 (4.6%)			
1–3 years	211 (14.0%)			
3–5 years	165 (11.0%)			
5–10 years	301 (20.0%)			
>10 years	744 (49.4%)			

## Measures

The questionnaire included demographic items (age, gender, occupation, education level, and work experience) alongside the following validated psychometric instruments.

*Emotional demands* (6 items) were assessed using the scale developed by Bakker et al. [8], and later presented in Xanthopoulou et al. [9], capturing both employees’ subjective perceptions of emotional demands (e.g., “Is your work emotionally demanding?”; “Do you face emotionally charged situations in your work?”), and their experiences of emotionally demanding interactions in the work environment (e.g., “In your work, do you deal with clients who incessantly complain, although you always do everything to help them?”; “Do you have to deal with clients who do not treat you with the appropriate respect and politeness?”). Responses were given on a 5-point Likert scale ranging from 1 (never) to 5 (always). The scale was translated into Swedish by the research group responsible for the broader project, and then back-translated into English by a native English speaker. The final formulations were reviewed and refined through group discussions to ensure conceptual clarity and linguistic accuracy.

*Burnout* (16 items) was assessed using the Swedish version of the Oldenburg Burnout Inventory (OLBI), adapted by Peterson et al. [37]. The OLBI has been widely validated and adapted into multiple languages, demonstrating strong reliability and construct validity across cultural contexts. The Swedish adaptation by Peterson et al. [37] is among these, providing a robust instrument for assessing burnout in Swedish-speaking populations. The OLBI captures two core dimensions of burnout: exhaustion and disengagement, each measured with eight items, divided into four positively and four negatively worded statements. The exhaustion scale reflects physical, cognitive, and emotional fatigue (e.g., “There are days when I feel tired before I arrive at work”; “I can tolerate the pressure of my work well”), while the disengagement scale reflects psychological distancing from one’s work, work objects, or work content (e.g., “Lately, I tend to think less at work and do my job almost mechanically”; “I always find new and interesting aspects in my work”) [5]. Responses were given on a 4-point Likert scale ranging from 1 (strongly disagree) to 4 (strongly agree).

*Deep acting* (3 items) was assessed using the deep acting subscale of the Emotional Labour Scale developed by Brotheridge and Lee [38]. This scale assesses the extent to which individuals make an effort to genuinely feel and express the emotions required in their professional roles (e.g., “Try to actually experience the emotions that I must show”). Participants rated each item on a 5-point Likert scale, ranging from 1 (never) to 5 (always). The scale was translated into Swedish by the research group responsible for the broader project, then back-translated into English by a native English speaker and reviewed and refined through group discussions. While the original scale contains three items, one item was excluded during the measurement model evaluation based on its psychometric performance, resulting in a two-item operationalization of deep acting.

The work resources of *meaning of work*, *control* (influence at work), and *social support* at work were assessed using corresponding dimensions from the Copenhagen Psychosocial Questionnaire Version III (COPSOQ III) [17–18]. The *meaning of work* dimension captures the subjective experience of meaningfulness and the perceived importance of one’s work through three items (e.g., “Is your work meaningful?”). The *work control* (influence at work) dimension assesses employees’ perceived control over their tasks and decisions through four items (e.g., “Do you have any influence on what you do at work?”; “Do you have a say in choosing who you work with?”). The *social support* dimension captured both instrumental help and emotional support related to work issues (e.g., “If needed, do you receive support from your colleagues/supervisors?”; “Do your colleagues/supervisors listen to your work-related problems?”) on four items. Responses were rated on a 5-point Likert scale.

### Statistical analysis

We used Partial Least Squares Structural Equation Modelling (PLS-SEM), implemented in SmartPLS 4 (version 4.1.1.2) [36], to examine the hypothesized direct relationships between emotional demands and burnout, test the moderating effects of personal and organizational resources, and explore age as a three-way moderator on significant interaction pathways. PLS-SEM was chosen over covariance-based SEM (CB-SEM) because the model combined reflective and formative constructs, included multiple interaction and exploratory three-way moderation effects, and focused on explaining variance in burnout rather than testing overall model fit. While the direct associations between emotional demands and burnout were theory-driven, the moderation and age-related effects were partly exploratory, making PLS-SEM more suitable than CB-SEM for the present analysis [39–41]. Constructs were classified as reflective or formative based on their theoretical foundations and measurement logic [40–41]. Burnout (exhaustion and disengagement), deep acting, and resources (meaning of work, control, social support) were modelled as reflective constructs, representing latent psychological states expressed through correlated indicators. In contrast, emotional demands were modelled as a formative construct, composed of distinct indicators that jointly define the overall construct.

Measurement model evaluation for the formative constructs included collinearity diagnostics (Variance Inflation Factor, VIF), and significance and relevance of outer weights and loadings. For reflective constructs, we assessed internal consistency (Cronbach’s alpha, composite reliability), indicator reliability (outer loadings), convergent validity (average variance extracted, AVE), and discriminant validity (heterotrait-monotrait ratio, HTMT), following guidelines by Hair et al. [41]. The structural model was assessed for collinearity (VIF) and by bootstrapping (5,000 subsamples, bias-corrected two-tailed confidence intervals, α = .05) to determine the significance and strength of path coefficients (standardized *β*), explanatory power (*R²*), and effect sizes (*f²*) [42]. This approach allowed for testing direct and interaction effects while ensuring both construct validity and theoretical consistency. Missing data were minimal. Among the variables included in the analyses, one response was missing for Disengagement 3, five responses were missing for Deep Acting 2, and one response was missing for Deep Acting 1. Missing values were handled using the SmartPLS 4 default imputation procedure [36,41]. To reduce potential sources of bias, validated instruments were used, translation procedures included back-translation to ensure conceptual accuracy, and clear eligibility criteria were applied to define the study population.

## Results

### Measurement model evaluation

The formative construct Emotional Demands was modelled using two dimensions: Contact-Related Emotional Demands and Perceived Emotional Demands. All indicators showed acceptable levels of collinearity, with VIF values ranging from 1.550 to 2.039, well below the threshold of 5 suggested by Hair et al. [41]. While two indicators (Perceived ED2 and Contact ED5) exhibited non-significant outer weights (p = 0.271 and p = 0.76, respectively), their outer loadings were significant, supporting their absolute contribution to the construct. In line with PLS-SEM guidelines [41], these indicators were retained to preserve the content validity and ensure comprehensive coverage of the construct domain.

The evaluation of the reflective measurement model began with an assessment of indicator reliability via outer loadings. Items with very low loadings were removed, including Disengagement 7 (“I find my work to be a positive challenge”, loading = 0.391) and Deep Acting 6 (“Really try to feel the emotions I have to show as part of my job.”, loading = –0.438). After removing these, the model was re-estimated and Average Variance Extracted (AVE) was checked for each construct. While most constructs met the AVE threshold of 0.50, Disengagement continued to signal issues (AVE = 0.478). To address this, an additional weak item (Disengagement 13 “This is the only type of work that I can imagine myself doing”, loading = 0.531) was removed, which improved the AVE to an acceptable level (AVE = 0.520) (see Table 2). Convergent validity was thereby confirmed, as all constructs surpassed the 0.50 AVE threshold [41], indicating that each latent variable explains more than half of the variance of its indicators.

**Table 2:** Construct-level metrics and outer loadings.

Construct	Loadings	AVE	C’s α	*ρa*	*ρ* *c*
Control	0.709–0.805	0.579	0.759	0.773	0.846
Deep Acting	0.780–0.944	0.749	0.691	0.889	0.855
Disengagement	0.656–0.819	0.520	0.814	0.820	0.866
Exhaustion	0.537–0.823	0.509	0.858	0.869	0.891
Meaning	0.830–0.882	0.739	0.829	0.881	0.894
Support	0.762–0.861	0.664	0.832	0.842	0.887

*Note*: Thresholds for evaluating construct reliability and validity: Cronbach’s alpha and composite reliability (*ρ*_*a*_ and *ρ*_*c*_) > 0.70 to demonstrate acceptable internal consistency reliability and average variance extracted (AVE) ≥ 0.50 to confirm convergent validity [41].

Internal consistency reliability was then evaluated using Cronbach’s Alpha (CA), Composite Reliability (CR/RhoC), and Dijkstra–Henseler’s RhoA. All constructs demonstrated acceptable reliability. Composite reliability (CR) values exceeded the recommended threshold of 0.70 for all constructs [41]. Although Deep Acting showed a Cronbach’s alpha (CA = 0.691) slightly below the conventional threshold of > 0.70 [41], its composite reliability (CR = 0.855) and average variance extracted (AVE = 0.749) remained well above recommended levels, supporting the construct’s reliability and convergent validity [41] (see Table 2).

Finally, discriminant validity was assessed using the Heterotrait-Monotrait Ratio (HTMT). All construct pairs exhibited HTMT values well below the conservative threshold of 0.85, confirming discriminant validity across the reflective constructs (see Table 3).

**Table 3:** HTMT Matrix (Discriminant Validity).

	Control	Deep Acting	Disengagement	Exhaustion	Meaning	Support
**Control**	—	0.132	0.561	0.573	0.298	0.494
**Deep Acting**		—	0.212	0.083	0.220	0.110
**Disengagement**			—	0.708	0.762	0.540
**Exhaustion**				—	0.388	0.530
**Meaning**					—	0.369
**Support**						—

*Note*: HTMT values < 0.90 indicate acceptable discriminant validity [41].

### Structural model evaluation and hypothesis testing

**Fig 2:**
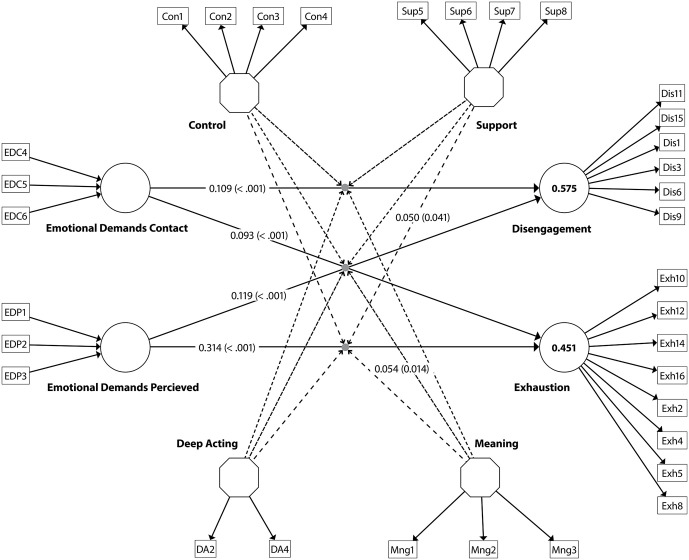
The structural model with the significant hypothesized paths.

The structural model was evaluated following the guidelines for PLS-SEM [41]. First, collinearity among predictor constructs was assessed using inner VIF values, all of which ranged from 1.05 to 1.52, well below the threshold of 5 [41], indicating no multicollinearity concerns. The model explained 57.5% (*R*^*2*^ *=* 0.575; adjusted *R*^*2*^ *=* 0.571) of the variance in disengagement and 45.1% (*R*^*2*^ *=* 0.451; adjusted *R*^*2*^ *=* 0446) in exhaustion, indicating moderate to substantial explanatory power, even when accounting for model complexity. The structural model with the significant hypothesized paths is presented in Fig 2.

Results are summarized in Table 4. All hypothesized direct effects from emotional demands to burnout outcomes were statistically significant. Specifically, contact-related emotional demands had a significant but small positive effect on disengagement (*β =* 0.109, *p* < .001), with a small effect size (*f²* = 0.023, *p* = .007), confirming H1a. Likewise, the effect on exhaustion was significant but small (*β =* 0.093, *p* < .001), with a small effect size (*f²* = 0.013, *p* = .037), confirming H1b. Perceived emotional demands had a significant but small positive effect on disengagement (*β =* 0.119, *p* < .001), with a small effect size (*f²* = 0.028, *p* = .003), confirming H2a. The effect of exhaustion was significant and moderate (*β =* 0.314, *p* < .001), with a moderate effect size (*f²* = 0.150, *p < .001*), confirming H2b.

**Table 4:** Path coefficients, significance, effect sizes, and variance inflation factor values.

Path	*β*	*p*	*f²*	VIF	Hypotheses
Direct Effects					
ED Contact → Disengagement	0.109	**< .001**	0.023	1.205	**H1a**
ED Contact → Exhaustion	0.093	**< .001**	0.013	1.205	**H1b**
ED Perceived → Disengagement	0.119	**< .001**	0.028	1.194	**H2a**
ED Perceived → Exhaustion	0.314	**< .001**	0.150	1.194	**H2b**
Deep Acting → Disengagement	−0.043	**0.018**	0.004	1.057	
Deep Acting → Exhaustion	−0.003	0.879	< .001	1.057	
Meaning → Disengagement	−0.531	**< .001**	0.536	1.236	
Meaning → Exhaustion	−0.195	**< .001**	0.056	1.236	
Control → Disengagement	−0.189	**< .001**	0.062	1.354	
Control → Exhaustion	−0.224	**< .001**	0.067	1.354	
Support → Disengagement	−0.148	**< .001**	0.039	1.318	
Support → Exhaustion	−0.202	**< .001**	0.056	1.318	
Moderation Effects					
Deep Acting × ED Contact → Disengagement	0.007	0.722	< .001	1.145	H3a
Deep Acting × ED Contact → Exhaustion	0.016	0.458	< .001	1.145	H3b
Deep Acting × ED Perceived → Disengagement	0.010	0.594	< .001	1.153	H3c
Deep Acting × ED Perceived → Exhaustion	−0.000	0.999	< .001	1.153	H3d
Meaning × ED Contact → Disengagement	0.035	0.084	0.003	1.309	H4a
Meaning × ED Contact → Exhaustion	0.054	**0.014**	0.005	1.309	H4b
Meaning × ED Perceived → Disengagement	0.033	0.096	0.002	1.377	H4c
Meaning × ED Perceived → Exhaustion	0.020	0.407	0.001	1.377	H4d
Control × ED Contact → Disengagement	−0.023	0.309	0.001	1.483	H5a
Control × ED Contact → Exhaustion	−0.009	0.705	< .001	1.483	H5b
Control × ED Perceived → Disengagement	−0.009	0.646	< .001	1.519	H5c
Control × ED Perceived → Exhaustion	0.019	0.441	0.001	1.519	H5d
Support × ED Contact → Disengagement	−0.009	0.658	< .001	1.377	H6a
Support × ED Contact → Exhaustion	−0.008	0.724	< .001	1.377	H6b
Support × ED Perceived → Disengagement	0.022	0.276	0.001	1.374	H6c
Support × ED Perceived → Exhaustion	0.050	**0.041**	0.004	1.374	H6d

*Note: β* = standardized beta coefficient; *f*² = effect size; *VIF* = variance inflation factor; *p* = significance level. Bolded values indicate statistically significant results (*p* < .05) and paths that support the hypothesized relationships.

Contrary to the JD-R-based buffering assumptions, two statistically significant moderation effects emerged, both in the opposite direction than hypothesized. Meaning of work showed a statistically significant but small positive interaction with contact-related emotional demands in predicting exhaustion (*β* = 0.054, *p* = .014). Although the associated effect size was negligible and non-significant (*f*² = 0.005, *p* = .245), the direction of the interaction was contrary to expectations, indicating a pattern of amplification rather than buffering, and thus H4b was not supported. Similarly, social support showed a statistically significant but small positive interaction with perceived emotional demands in predicting exhaustion (*β* = 0.050, *p* = .041), again with a negligible and non-significant effect size (*f*² = 0.004, *p* = .317). The direction of this interaction was also opposite to the hypothesized buffering effect, and H6d was, therefore, not supported. No other moderation effects were statistically significant (all *p* > .05).

Although the primary focus was on the moderating role of job resources, the model also revealed several direct associations between these resources and burnout dimensions. Deep acting showed a statistically significant but small negative association with disengagement (*β =* –0.043, *p* = .018), accompanied by a negligible and non-significant effect size (*f²* = 0.004, *p* = .270), and no significant association with exhaustion (*β =* –0.003, *p* = .879; *f²* = < .001, *p* = .987), indicating a limited direct role of this emotion regulation strategy in burnout. In contrast, meaning of work showed a strong negative association with disengagement (*β =* –0.531, *p* < .001; *f²* = 0.536, *p* < .001), and a moderate negative association with exhaustion (*β =* –0.195, *p* < .001; *f²* = 0.056, *p* < .001). Control was also negatively associated with disengagement (*β =* –0.189, *p* < .001), and exhaustion (*β =* –0.224, *p* < .001) with small but meaningful effect sizes (*f²* = 0.062, *p* < .001, and *f²* = 0.067, *p* < .001 respectively). Similarly, support was negatively associated with disengagement (*β =* –0.148, *p* < .001; *f²* = 0.039, *p* = .001) and exhaustion (*β =* –0.202, *p* < .001; *f²* = 0.056, *p* < .001). Overall, these findings indicate that meaning of work, control, and social support are associated with lower general levels of burnout. However, taken together with the moderation results, these resources show differential effects under the conditions of high emotional demands, highlighting a distinction between their general protective role and their function in emotionally demanding work contexts.

### Exploratory age analyses

To assess whether the moderating effects of job resources vary by age, we re-estimated the structural model by adding two three-way interaction terms involving (standardized) age. These were specified only for the two significant moderator paths, Meaning × Contact-related Emotional Demands, and Support × Perceived Emotional Demands, both related to exhaustion. No additional three-way interaction terms were added. The inclusion of three-way interaction terms with standardized age revealed no significant moderation effects. Neither the interaction of age, support, and perceived emotional demands (*β =* –0.019, *p =* 0.350), nor the interaction of age, meaning, and contact-related emotional demands (*β =* 0.006, *p =* 0.777) was significant in predicting exhaustion. These results suggest that the moderating influence of job resources on the relationship between emotional demands and exhaustion does not vary meaningfully by age.

Although the three-way interaction terms involving age were non-significant, we conducted a follow-up analysis to examine whether age may have a direct effect on burnout. While age is frequently associated with lower burnout in previous research, the underlying mechanisms remain unclear. It is possible that age-related effects are not fully explained by the job resources examined in this model (i.e., control, support, meaning, and deep acting), and that other factors may account for age-related differences in emotional exhaustion and disengagement. To test this, standardized age was added as a direct predictor of both burnout dimensions. Indeed, the results indicated that age had no significant direct effect on either outcome, Exhaustion (*β =* –0.036, *p =* 0.078) or Disengagement (*β =* –0.029, *p =* 0.116).

To further explore whether age might influence the moderating effects of job resources in a nonlinear (curvilinear) way and capture possible U-shaped or inverted U-shaped effects of age, we re-estimated the structural model by adding two three-way interaction terms involving squared standardized age (Z_Age²). These three-way interaction terms were specified only for the two previously significant moderation paths: (a) Meaning × Contact-related Emotional Demands, and (b) Support × Perceived Emotional Demands, both predicting exhaustion. The results revealed no significant curvilinear moderation effects. Neither the interaction of curvilinear age, support, and perceived emotional demands (*β =* –0.021, *p =* 0.336), nor the interaction of curvilinear age, meaning, and contact-related emotional demands (*β =* 0.004, *p =* 0.858) was significant in predicting exhaustion. These findings suggest that the moderating influence of job resources on the emotional demands–burnout relationship does not vary meaningfully across curvilinear patterns of age in this sample.

To test for curvilinear relationships between age and burnout, standardized age squared (Z_Age²) was added as a predictor of exhaustion and disengagement. Z_Age² was not significantly associated with exhaustion (*β =* –0.021, *p =* 0.278), indicating no curvilinear age effect on the exhaustion dimension. For disengagement, a small but significant curvilinear effect was found (*β =* 0.045, *p =* 0.013). However, the corresponding effect size was small and non-significant (*f*² = 0.005, *p* = .237), suggesting that the practical relevance of this effect is negligible.

## Discussion

This study investigated how perceived and contact-related emotional demands at work relate to the burnout dimensions of disengagement and exhaustion, and whether the job resources of deep acting, meaning of work, control, and social support moderate these relationships. All hypothesized direct effects were supported. Among the moderators, meaning of work and social support significantly influenced the relationship between emotional demands and exhaustion. However, contrary to expectations, both effects were positive, indicating that higher levels of these resources intensified rather than buffered the impact of emotional demands. No other moderating effects were statistically significant. At the same time, several resources showed consistent negative associations with burnout. Finally, exploratory analyses involving age revealed no significant linear or curvilinear moderation effects. A small U-shaped association between age and disengagement was observed, but its negligible effect size indicates limited practical relevance.

These findings address an important gap in the Job Demands–Resources (JD–R) literature by showing that job resources, including deep acting, meaning of work, control, and social support, may not uniformly buffer against burnout in emotionally demanding work contexts. While the JD–R model assumes that resources mitigate the impact of job demands, the present results indicate that commonly studied resources did not buffer these relationships and that meaning of work and social support were associated with an amplification of the effects of emotional demands on exhaustion. This highlights the need for a more context-sensitive understanding of how job resources operate under conditions of sustained emotional strain.

### Emotional demands and burnout

This study demonstrated that both perceived and contact-related emotional demands were positively associated with the burnout dimensions of exhaustion and disengagement, confirming Hypotheses 1a, 1b, 2a, and 2b. These findings are consistent with the Job Demands–Resources (JD-R) model, which posits that high job demands contribute directly to strain outcomes such as burnout [4–7]. Notably, perceived emotional demands had the strongest relationship with exhaustion, compared to both disengagement and the burnout relationships involving contact-related emotional demands. While both perceived and contact-related demands were significantly associated with burnout, the stronger link between perceived emotional demands and exhaustion aligns with research suggesting that subjectively appraised emotional strain more directly reflects the chronic psychological burden of emotionally demanding work [9,43]. Perceived emotional demands may therefore capture more than discrete emotionally intense encounters, reflecting the cumulative burden or exposure to a wider array of emotionally taxing stimuli within the work environment, as well as sustained efforts of regulating emotions at work. In sum, these findings confirm the central role of emotional demands in driving burnout and highlight the importance of considering both workers’ subjective perceptions and contact-related exposures when examining emotional demands.

### Rethinking resources: When buffering fails

While the JD-R model positions workplace-related and personal resources as protective factors, the present findings suggest that this assumption may not hold in the context of emotionally demanding work, where some resources fail to buffer strain and may rather intensify burnout under high emotional demands. Contrary to Hypotheses H3a-d, deep acting did not moderate the relationship between emotional demands and burnout. Although deep acting did not exacerbate strain, it also failed to buffer it. This suggests that deep acting, while generally regarded as a more adaptive emotional labour strategy compared to surface acting (see Hülsheger and Schewe [21] for a review), did not function as a protective personal resource in this study. Deep acting may reduce emotional dissonance and foster authenticity in interpersonal encounters, but it may still require effort [44]. Prior research suggests that although deep acting is less harmful than surface acting, it does not consistently protect against emotional exhaustion in high-demand contexts [44,45]. Mann et al. [46] further argue that the effectiveness of deep acting may depend on contextual and individual factors, including the degree of alignment between an individual’s values and the emotional display rules of the job. When this alignment is weak, even deep acting may feel effortful or inauthentic, diminishing its protective qualities. Thus, while deep acting may be more constructive and less straining than other emotion regulatory strategies (e.g., surface acting or suppression), it may not serve as a sustainable resource capable of buffering burnout under conditions of persistent emotional demands.

Similarly, contrary to Hypotheses 4a–d, meaning of work did not moderate the relationship between emotional demands and burnout in the expected direction. Instead, when contact-related emotional demands were high, the perceived meaningfulness of one´s work intensified exhaustion. This amplification effect represents a particularly important departure from core JD-R assumptions, suggesting that meaning may increase vulnerability, rather than resilience under conditions of sustained or high emotional demands. One possible explanation is that employees who view their work as deeply meaningful may internalize emotional demands as being central to their professional identity, particularly in human contact professions. When employees equate delivering emotionally responsive service with being a “good professional,” they may push themselves beyond their emotional limits to uphold these ideals. This overextension, when not supported by adequate structural resources, may lead to emotional depletion rather than resilience. Indeed, meaningful work has been characterized as a “double-edged sword” that can both alleviate and exacerbate stress depending on the context [47]. Caring deeply about one’s work may increase perceived responsibility and heighten self-imposed expectations, thereby intensifying strain when emotional demands are high [47–49]. When work matters deeply, failures or constraints can feel more personally significant, turning external demands into self-relevant threats. In this way, meaning at work may not always serve a protective function; instead, it may increase stress [47,49] and potentially heighten vulnerability to burnout by amplifying the psychological toll of emotionally demanding work.

Contrary to Hypotheses H5a–d, control did not moderate the relationship between emotional demands and burnout. Although work control, typically defined as decision latitude or influence over one’s work, is widely regarded as a protective factor in occupational stress models [50], its effects may be context-dependent. In emotionally demanding roles, high levels of control may not mitigate strain and could even place greater responsibility on the individual to manage emotional stressors independently. Dettmers and Bredehöft [51] similarly note that autonomy can entail job design demands, that is, the requirement to self-organize, plan, and optimize one’s work, which can result in that control itself becoming an additional demand. When employees are expected to handle emotional demands on their own, autonomy may intensify the psychological burden by reinforcing a sense of personal accountability for outcomes that are structurally constrained. Workers might feel that they should be capable of coping with emotional challenges due to their autonomy, and when they cannot, they may internalize this gap as personal failure. Moreover, emotional demands are often underacknowledged within organizational cultures and may lack clear structural boundaries. Without explicit recognition or regulation of what constitutes acceptable emotional strain, workers may be left to manage these demands alone, rendering control at work as an insufficient protective resource.

Neither did social support buffer the impact of emotional demands on burnout, contrary to H6a-d. Notably, and in line with the findings for meaning of work, social support showed an amplification effect, intensifying the effect of perceived emotional demands on exhaustion. While social support at work is widely regarded as a protective job resource in the JD-R model [7], meta-analytic evidence shows that its effects are inconsistent and depend strongly on the source of support [52]. One explanation is that support in demanding work contexts may be activated reactively rather than proactively, functioning less as a preventive buffer and more as a signal that distress has already accumulated. For instance, AbuAlRub [53] found that while coworker support was associated with lower stress and higher performance among nurses, it did not significantly buffer the stress–performance relationship, suggesting that support often comes too late to offset ongoing strain. Moreover, the content of supportive interactions plays a critical role. Maladaptive forms of support, such as venting or co-rumination may even heighten exhaustion. As Boren [54] demonstrated, co-rumination suppresses the buffering effect of support on emotional exhaustion, as excessive problem-focused talk fosters emotional contagion and amplifies stress. Finally, in highly interdependent work environments such as healthcare, strong social cohesion can foster implicit pressures toward resilience and self-sacrifice. As Leiter and Maslach [55] note, nurses often feel obliged to overextend themselves to maintain patient care and team functioning, even under unsustainable demands. In such contexts, social support may help sustain performance and organizational delivery, but it does so at the cost of individual well-being.

Together, these findings highlight the need to re-evaluate how organizational and personal resources function in emotionally demanding work contexts. Rather than acting as universal buffers, some widely valued resources may, under certain conditions, amplify vulnerability, particularly when they foster overcommitment, overidentification with one’s role, or reactive rather than proactive coping strategies. This underscores the importance of exploring not only which resources buffer the relationship between emotional demands and burnout, but also how they function, that is, examining the psychological mechanisms through which resources exert their effects, as well as distinguishing between resource availability (whether a resource is present) and resource utility (whether it is accessed and used effectively and adaptively within a given context).

### Direct effects of job resources

Consistent with the core assumptions of the JD-R model, the job resources of meaning of work, work control, and social support showed moderate to strong negative direct associations with both exhaustion and disengagement, while deep acting had a weaker but statistically significant negative association with disengagement (and no association with exhaustion). These findings align with the central proposition of the Job Demands–Resources (JD-R) model, namely that workplace-related and personal resources promote employee well-being and resilience by fostering motivation and reducing the likelihood of strain [4,7,20]. Specifically, job resources are thought to support psychological functioning by enhancing work engagement, facilitating goal achievement, and reducing the impact of demands over time [8,19]. However, when considered alongside the moderation results in our study, a more differentiated pattern emerges. While these resources are associated with lower overall burnout, they did not buffer and, in some cases, rather amplified the impact of emotional demands. This may indicate that emotional demands, as a distinct and often interpersonal form of stressor, require more targeted or specialized resources, for example, psychological flexibility, or structural and institutional regulations, to be adequately mitigated.

### Exploratory age analyses

The exploratory analyses indicated that age did not influence how resources moderated the effects of emotional demands. Specifically, the three-way interaction terms involving age and the two significant moderator paths were non-significant, both when age was modelled as a linear variable and as a curvilinear term. This suggests that age does not meaningfully influence how the resources of meaning of work or social support at work affect the relationship between emotional demands and exhaustion. In other words, there was no evidence that younger, middle-aged, or older employees differ in how they utilize or benefit from these resources in emotionally demanding contexts.

Further, age did not show a significant linear association with exhaustion or disengagement. However, a small curvilinear (U-shaped) association was found for disengagement, indicating slightly higher disengagement levels among both younger and older workers, with lower disengagement in the middle-age range. Although the effect size was negligible and should be interpreted cautiously, this pattern may tentatively reflect transitional phases across the working life course. Prior research has similarly suggested non-linear trajectories in burnout and work-related attitudes, with less favourable outcomes in early and late working life and more favourable outcomes in mid-career [56–58]. Such patterns have been attributed to career uncertainty in early working life and gradual psychological disengagement toward later career stages, whereas mid-career employees tend to benefit from greater role clarity, stability, and organizational embeddedness, which contribute to stronger career commitment and lower disengagement.

### Strengths and limitations

This study offers several methodological and theoretical strengths. First, it employed PLS-SEM, which enabled the simultaneous estimation of complex models with multiple formative and reflective latent constructs, including the evaluation of interaction and moderation effects. The measurement models were rigorously assessed to ensure construct validity, and all hypothesized relationships were systematically tested and transparently reported. The study also included a relatively large and multi-professional sample, enhancing the robustness and practical relevance of the findings across a diverse occupational context. Additionally, this study extended the literature by exploring potential age-related effects, including both linear and curvilinear patterns as well as three-way interactions involving age, thereby addressing underexamined dynamics in the emotional demands and burnout relationship. However, several limitations should be noted. First, the cross-sectional design of the study precludes causal inferences, making it impossible to determine the temporal ordering of emotional demands, job resources, and burnout outcomes, or to examine how these relationships unfold over time. Second, the strong predominance of women in the sample (93.4%) constitutes a clear limitation and restricts the generalizability of the findings to male participants and more gender-balanced or male-dominated occupational contexts. At the same time, this gender distribution reflects the composition of Sweden’s healthcare, care, and service sectors [59], which supports ecological validity within these occupational contexts. In addition, because participation was voluntary and recruitment relied primarily on social media, self-selection bias cannot be ruled out, as individuals with an interest in or experience of emotional demands at work may have been more likely to participate. Moreover, this recruitment strategy did not allow for estimation of non-response rates, which limits assessment of potential non-response bias. Third, the study included only a subset of job demands and resources, meaning that other factors are likely to shape burnout, but were not accounted for. Fourth, age was measured only chronologically, which limits the ability to assess subjective or functional dimensions of aging (e.g., work tenure, or life stage-related factors) that may influence emotional regulation or resource availability and use. Fifth, although the deep acting construct met acceptable psychometric criteria, it may be insufficiently captured in the present study, as only two out of three original items were retained, and its association with burnout was weak. While item reduction, including the use of few-item or even single-item constructs, is acceptable in PLS-SEM, and can be necessary to enhance construct validity within a given sample, this approach reduces direct comparability with prior research using full scales and limits the breadth of the deep acting construct. In contrast, the burnout dimensions were more robustly measured with multiple retained indicators; nevertheless, item-level modifications should be considered when comparing findings across studies. Finally, the reliance on self-report data collected at a single time point raises the potential for common method bias (CMB) [60], which could inflate associations due to shared method variance. While the use of PLS-SEM and a well-differentiated measurement model may help reduce this risk, future studies should consider incorporating multi-source or time-lagged designs to mitigate CMB concerns.

### Implications

This study underscores the complexity of how emotional demands and job resources interact in shaping burnout outcomes. While job resources such as work meaning, autonomy, and social support were associated with lower general levels of exhaustion and disengagement, they offered limited buffering when emotional demands were high. Unexpectedly, higher levels of meaning and support amplified the relationship between emotional demands and exhaustion, suggesting that in emotionally taxing roles, these resources may not always function protectively. This challenges assumptions within the Job Demands–Resources (JD–R) model and highlights the need to consider not only the presence of resources, but also how they are used and embedded in emotionally demanding contexts.

From a practical perspective, these findings suggest that simply increasing individual-level resources may be insufficient to mitigate burnout in emotionally demanding work. While interventions to enhance workers’ emotion regulation capabilities, as well as foster meaning, support, and autonomy, remain important, they may need to be complemented with more systemic organizational strategies that address the emotional aspects of the work more directly. For example, adjusting contact frequency or workload in emotionally demanding roles, providing structured individual or team-based reflective debriefing, creating space to acknowledge emotional strain, setting clearer expectations around emotional requirements, as well as value-based emotion regulation training, or targeted coaching might help organizations address emotional demands more directly, rather than relying solely on individual coping resources.

An important insight from this study is that interventions in emotionally demanding professions may need to go beyond strengthening job resources alone and consider how emotional demands themselves are structured and managed. While resources such as deep acting, meaning of work, control, and social support were associated with lower overall burnout, they did not appear to buffer the effects of emotional demands and, in the cases of meaning of work and social support, were associated with higher exhaustion when emotional demands are high. This his suggests that organizations could benefit from complementing resource-based approaches with strategies that more directly address emotional demands, for example by examining how such demands arise in everyday work and how employees navigate them.

### Future directions

Despite extensive research on the role of resources in mitigating burnout, less is known about why some resources may, under certain conditions, amplify rather than buffer strain. This gap highlights the need to identify boundary conditions under which resources switch roles. To address this, future research could examine mechanisms such as professional role identity, emotional overcommitment, or maladaptive support-seeking patterns (e.g., venting or co-ruminating). Expanding the model to include additional personal or organisational resources also warrants investigation to identify more effective buffers against emotional strain. Potential personal resources may include psychological flexibility or emotional intelligence, while organizational resources could involve psychological safety, psychosocial safety climate, and opportunities for reflection or recovery. Longitudinal and mixed-methods designs may be particularly valuable for capturing temporal and contextual fluctuations in resource use and burnout symptoms, allowing future research to better examine causal processes and how burnout develops over time. In addition, future studies could integrate physiological indicators (e.g., heart rate variability or cortisol levels) and organizational-level measures (e.g., objective workload indicators) to complement self-report data and reduce common method bias. While the present study did not find significant moderation effects by age, future work might examine how career stage, specific professional roles (e.g., managers, supervisors), or long-term emotional exposure (accounting for early career leave) influence access to and use of resources in emotionally demanding roles. Finally, increasing diversity in terms of gender representation would enhance the generalizability of findings.

## Conclusion

This study confirmed that both perceived and contact-related emotional demands have a significant positive relationship with the burnout dimensions of disengagement and exhaustion, supporting all hypothesized direct effects. While job resources such as work meaning, control, and social support were associated with lower overall burnout levels, they did not buffer the negative effects of emotional demands on burnout as anticipated. On the contrary, meaning of work and social support at work amplified the relationship between emotional demands and exhaustion. These findings challenge the assumption that job resources universally protect against strain and highlight the importance of understanding how resources interact with the psychological mechanisms involved in emotionally demanding work. Finally, age played a limited role, showing no significant moderating effects and only a negligible curvilinear association with disengagement. Taken together, these results call for a reconsideration of core JD–R assumptions under the conditions of emotional demands, emphasizing the need for more nuanced, context-sensitive models of how resources function in emotionally demanding professions.
