# Supplementary material for: Emotional demands and burnout: Differential moderating effect of job resources and age
Source: PLoS One. 2026 Jun 22;21(6):e0351940. doi: 10.1371/journal.pone.0351940 (PMC13286175; doi:10.1371/journal.pone.0351940)
Supplement: Attachment — Submitted filename: Response to Reviewers..docx [file pone.0351940.s003.docx]

**Reviewer #2**

We thank the reviewer for their positive evaluation of the manuscript and for acknowledging the revisions made. We are pleased that the reviewer is satisfied with the current version.

**Reviewer #3**

We thank the reviewer for their thoughtful and constructive comments, as well as for the positive feedback on the revisions made in the previous round. We appreciate the opportunity to further strengthen the manuscript and have addressed each of the points raised below.

Comment:

“- Please share the EQUATOR guidelines to report the rigor of this study. this could be attached as a supplement as well.”

Response:

Thank you for this valuable suggestion. To enhance transparency and reporting rigor, we have ensured that the manuscript adheres to the STROBE guidelines for cross-sectional studies. A completed STROBE checklist has been added as Supplementary Material (S1 File), indicating where each reporting item is addressed in the manuscript. In addition, a statement has been included in the Methods section (lines 222-224) to clarify that the study is reported in accordance with these guidelines.

Comment:
“-it would be a great asset to state that how the findings of the study could be help bridge the gap of the study which could come out strongly in the manuscript as well and probably would benefit from a figure too.”

Response:
Thank you for this insightful suggestion. To make the theoretical contribution more explicit, we have revised the opening of the Discussion section (lines 515–522) to more clearly articulate how the findings address a gap in the Job Demands–Resources (JD–R) literature, particularly by showing that job resources may not uniformly buffer the effects of emotional demands and may, in some cases, be associated with amplification effects. To avoid redundancy with the existing figures, we addressed this point through a strengthened conceptual explanation in the text rather than adding an additional figure.

Comment:

- Is there something as a strong outcome that could be implemented coming out from the study. This could be reflected well in discussion section.

Response:
Thank you for this helpful suggestion. To more clearly articulate the practical implications, we have expanded the Implications section (lines 737–746) to highlight that interventions in emotionally demanding professions may need to go beyond strengthening job resources alone and consider how emotional demands themselves are structured and managed. This addition clarifies how the findings can inform practice while maintaining an appropriately cautious interpretation.

--

We sincerely thank the Academic Editor and both reviewers for their valuable feedback, which has helped improve the clarity and contribution of the manuscript.
