# Supplementary material for: Emotional demands and burnout: Differential moderating effect of job resources and age
Source: PLoS One. 2026 Jun 22;21(6):e0351940. doi: 10.1371/journal.pone.0351940 (PMC13286175; doi:10.1371/journal.pone.0351940)
Supplement: Attachment — Submitted filename: Response to Reviewer_R3.docx [file pone.0351940.s004.docx]

Dear Dr. Hu,

We would like to thank you for your careful evaluation of our manuscript, Emotional demands and burnout: differential moderating effect of job resources and age [PONE-D-25-45860R2] - [EMID:1a52517e7f43393a], and for your constructive comments. We appreciate your positive assessment of the revisions made thus far and are grateful for the opportunity to further improve the manuscript. We would also like to thank Reviewer #2 for the careful evaluation of the manuscript and for the constructive feedback provided throughout the review process. We appreciate the reviewer’s time and effort in helping to improve the quality and clarity of this work.

In response to the additional editorial comments, we have carefully revised the manuscript and addressed each point raised. The changes made are detailed below.

Values currently reported as p = 0.000 should be changed to p < .001 or p < 0.001, as appropriate. Please ensure that this formatting is applied consistently in the text, tables, and figures.

**Response:**
Thank you for this observation. We have revised the reporting of p-values throughout the manuscript and replaced all instances of p = 0.000 with p < .001, as appropriate. These changes have been applied consistently in the text, tables, and figures.

Please check the formatting of statistical notation throughout the manuscript. For example, R² = 0,575 and adjusted R² = 0,571. These should be revised to decimal points, for example R² = 0.575 and adjusted R² = 0.571.

**Response:**
Thank you for this observation. We have reviewed the statistical notation throughout the manuscript and corrected all instances where decimal commas were used instead of decimal points. These corrections have been applied consistently in the text, tables, and figures, including Figure 2.

Please carefully verify the values reported for Deep Acting in Table 2. The reported loading range for Deep Acting appears difficult to reconcile with the reported AVE and composite reliability values. Please check whether the final retained item loadings, AVE, Cronbach’s alpha, ρa, and ρc are all taken from the same final measurement model.

**Response:**

Thank you for identifying this inconsistency. Upon re-examining the measurement model output, we found that the loading ranges reported in Table 2 corresponded to the initial item loadings rather than the final loadings obtained after model estimation. The AVE, Cronbach’s alpha, ρa, and composite reliability (ρc) values were correctly reported from the final measurement model. We have therefore updated the loading ranges for all constructs in Table 2 so that all reported metrics are derived consistently from the same final measurement model. This correction does not affect the results or conclusions of the study.

In the paragraph discussing the measurement model, please correct the cross-reference if needed. The text appears to refer to “Table 1” when the construct-level metrics are actually presented in Table 2.

**Response:**
Thank you for identifying this error. We have corrected the cross-reference in the manuscript. The paragraph discussing the measurement model now correctly refers to Table 2.

The text currently states that all constructs showed Cronbach’s alpha and composite reliability values above .70, but Deep Acting is reported with Cronbach’s alpha = .691.

**Response:**
Thank you for identifying this inconsistency. We have revised the text describing the reliability results to reflect the reported values accurately. The revised wording acknowledges that Deep Acting showed a Cronbach’s alpha slightly below the conventional threshold, while its composite reliability and average variance extracted remained above recommended levels.

The STROBE checklist notes that missing data are described but not reported per variable. Please either report the number of missing values for each variable.

**Response:**
Thank you for this observation. We have revised the Methods section to report the extent of missing data for the variables included in the analyses. Missing data were minimal, with one missing response for Disengagement 3, five missing responses for Deep Acting 2, and one missing response for Deep Acting 1. We also updated the STROBE checklist accordingly.

Please carefully proofread the Acknowledgments and final sections for minor typographical and punctuation errors. For example, there appear to be duplicated or incorrectly punctuated phrases such as “University of Gävle.Support,” “professions. .,” and “over time..”. These should be corrected before publication.

**Response:**
Thank you for identifying these issues. We carefully proofread the Acknowledgments and final sections of the manuscript and corrected typographical, spacing, and punctuation errors, including duplicated punctuation marks and formatting inconsistencies where present.

Please ensure that the final figure files and figure captions are consistent with the revised manuscript.

**Response:**
Thank you for this reminder. We reviewed all figure files and figure captions and verified that they are consistent with the revised manuscript. Where necessary, statistical notations were updated to ensure consistency with the text and tables. The final figures have been provided in EPS format in accordance with the journal’s publication requirements and in PNG format for viewing purposes.
