# Supplementary material for: Emotional demands and burnout: Differential moderating effect of job resources and age
Source: PLoS One. 2026 Jun 22;21(6):e0351940. doi: 10.1371/journal.pone.0351940 (PMC13286175; doi:10.1371/journal.pone.0351940)
Supplement: Peer review history

# Peer review: Decision Letter 0

**DOI:** 10.1371/journal.pone.0351940.r001

**Authors:** Ioana Gutu

11 Dec 2025

PONE-D-25-45860

Emotional demands and burnout: The moderating role of deep acting, meaning of work, work control, social support at work, and age

PLOS One

Dear Dr. Popucza,

Thank you for submitting your manuscript to PLOS ONE. After careful consideration, we feel that it has merit but does not fully meet PLOS ONE’s publication criteria as it currently stands. Therefore, we invite you to submit a revised version of the manuscript that addresses the points raised during the review process.

As for improving your manuscript, please carefully consider all the points raised by both reviewers, according to their position towards the paper.

Please submit your revised manuscript by Jan 25 2026 11:59PM. If you will need more time than this to complete your revisions, please reply to this message or contact the journal office at plosone@plos.org. When you're ready to submit your revision, log on to https://www.editorialmanager.com/pone/ and select the 'Submissions Needing Revision' folder to locate your manuscript file.

Please include the following items when submitting your revised manuscript:

- A rebuttal letter that responds to each point raised by the academic editor and reviewer(s). You should upload this letter as a separate file labeled 'Response to Reviewers'.
- A marked-up copy of your manuscript that highlights changes made to the original version. You should upload this as a separate file labeled 'Revised Manuscript with Track Changes'.
- An unmarked version of your revised paper without tracked changes. You should upload this as a separate file labeled 'Manuscript'.

If you would like to make changes to your financial disclosure, please include your updated statement in your cover letter. Guidelines for resubmitting your figure files are available below the reviewer comments at the end of this letter.

If applicable, we recommend that you deposit your laboratory protocols in protocols.io to enhance the reproducibility of your results. Protocols.io assigns your protocol its own identifier (DOI) so that it can be cited independently in the future. For instructions see: https://journals.plos.org/plosone/s/submission-guidelines#loc-laboratory-protocols. Additionally, PLOS ONE offers an option for publishing peer-reviewed Lab Protocol articles, which describe protocols hosted on protocols.io. Read more information on sharing protocols at https://plos.org/protocols?utm_medium=editorial-email&utm_source=authorletters&utm_campaign=protocols.

We look forward to receiving your revised manuscript.

Kind regards,

Ioana Gutu, Postdoctoral

Academic Editor

PLOS One

Journal Requirements:

When submitting your revision, we need you to address these additional requirements.

1. Please ensure that your manuscript meets PLOS ONE's style requirements, including those for file naming. The PLOS ONE style templates can be found at https://journals.plos.org/plosone/s/file?id=wjVg/PLOSOne_formatting_sample_main_body.pdf and https://journals.plos.org/plosone/s/file?id=ba62/PLOSOne_formatting_sample_title_authors_affiliations.pdf

2. We note that you have indicated that there are restrictions to data sharing for this study. For studies involving human research participant data or other sensitive data, we encourage authors to share de-identified or anonymized data. However, when data cannot be publicly shared for ethical reasons, we allow authors to make their data sets available upon request. For information on unacceptable data access restrictions, please see http://journals.plos.org/plosone/s/data-availability#loc-unacceptable-data-access-restrictions.

Before we proceed with your manuscript, please address the following prompts:

a) If there are ethical or legal restrictions on sharing a de-identified data set, please explain them in detail (e.g., data contain potentially identifying or sensitive patient information, data are owned by a third-party organization, etc.) and who has imposed them (e.g., a Research Ethics Committee or Institutional Review Board, etc.). Please also provide contact information for a data access committee, ethics committee, or other institutional body to which data requests may be sent.

b) If there are no restrictions, please upload the minimal anonymized data set necessary to replicate your study findings to a stable, public repository and provide us with the relevant URLs, DOIs, or accession numbers. Please see http://www.bmj.com/content/340/bmj.c181.long for guidelines on how to de-identify and prepare clinical data for publication. For a list of recommended repositories, please see https://journals.plos.org/plosone/s/recommended-repositories. You also have the option of uploading the data as Supporting Information files, but we would recommend depositing data directly to a data repository if possible.

Please update your Data Availability statement in the submission form accordingly.

3. In the online submission form, you indicated that the data underlying this study contain sensitive health information and are stored in a secure repository at the University of Gävle. Due to ethical and legal restrictions, the data cannot be made publicly available. Data may be made available from the corresponding author upon reasonable request and with permission of the University of Gävle.

All PLOS journals now require all data underlying the findings described in their manuscript to be freely available to other researchers, either 1. In a public repository, 2. Within the manuscript itself, or 3. Uploaded as supplementary information.

This policy applies to all data except where public deposition would breach compliance with the protocol approved by your research ethics board. If your data cannot be made publicly available for ethical or legal reasons (e.g., public availability would compromise patient privacy), please explain your reasons on resubmission and your exemption request will be escalated for approval.

4. If the reviewer comments include a recommendation to cite specific previously published works, please review and evaluate these publications to determine whether they are relevant and should be cited. There is no requirement to cite these works unless the editor has indicated otherwise.

[Note: HTML markup is below. Please do not edit.]

Reviewers' comments:

Reviewer's Responses to Questions

**Comments to the Author**

1. Is the manuscript technically sound, and do the data support the conclusions?

The manuscript must describe a technically sound piece of scientific research with data that supports the conclusions. Experiments must have been conducted rigorously, with appropriate controls, replication, and sample sizes. The conclusions must be drawn appropriately based on the data presented.

Reviewer #1: Partly

Reviewer #2: Yes

**********

2. Has the statistical analysis been performed appropriately and rigorously?

Reviewer #1: Yes

Reviewer #2: Yes

**********

3. Have the authors made all data underlying the findings in their manuscript fully available?

The PLOS Data policy requires authors to make all data underlying the findings described in their manuscript fully available without restriction, with rare exception (please refer to the Data Availability Statement in the manuscript PDF file). The data should be provided as part of the manuscript or its supporting information, or deposited to a public repository. For example, in addition to summary statistics, the data points behind means, medians and variance measures should be available. If there are restrictions on publicly sharing data—e.g. participant privacy or use of data from a third party—those must be specified.

Reviewer #1: No

Reviewer #2: Yes

**********

4. Is the manuscript presented in an intelligible fashion and written in standard English?

PLOS ONE does not copyedit accepted manuscripts, so the language in submitted articles must be clear, correct, and unambiguous. Any typographical or grammatical errors should be corrected at revision, so please note any specific errors here.

Reviewer #1: Yes

Reviewer #2: Yes

**********

5. Review Comments to the Author

Please use the space provided to explain your answers to the questions above. You may also include additional comments for the author, including concerns about dual publication, research ethics, or publication ethics. (Please upload your review as an attachment if it exceeds 20,000 characters)

Reviewer #1: Dear author,

I have carefully reviewed your manuscript, which addresses a highly relevant and timely issue. The paper is clearly structured and well written. At the same time, there are several areas where further refinement could enhance its clarity, theoretical contribution, and practical relevance. In what follows, I provide detailed feedback for each section with the intention of supporting the improvement of the manuscript.

Title

Strengths: Clear, informative, and well aligned with the study’s aims. It specifies the core variables and signals the moderating role of resources and age. Points for improvement: The length could be slightly reduced to increase readability. A more concise version may enhance impact, e.g., “Emotional Demands, Burnout, and the Moderating Role of Resources and Age.”

Abstract

Strengths: Provides a concise overview of theoretical basis (JD-R model), methods (sample, PLS-SEM), main findings, and contributions. Clear separation of direct, moderating, and exploratory effects. Points for improvement: The abstract is dense and may overwhelm readers. Simplifying some methodological details (e.g., bootstrapping, effect sizes) and emphasizing key contributions more directly would improve accessibility for a broad audience.

Keywords

Strengths: Relevant, aligned with the study’s constructs, and likely to optimize discoverability.

Points for improvement: Could benefit from including “JD-R model” explicitly, as it is central to the study.

Introduction

Strengths: Comprehensive literature review, solid grounding in JD-R, clear positioning of emotional demands, and good integration of personal/organizational resources and age.

Points for improvement: The knowledge gap is somewhat implicit. It would help to explicitly highlight how this study advances beyond existing research (e.g., inconsistent findings on moderation, limited evidence for three-way interactions with age).

Figure 1 (Conceptual model)

Strengths: Presents the hypothesized relationships clearly and follows JD-R conventions.

Points for improvement: The figure should be more visually differentiated (e.g., distinct arrows for direct vs. moderating effects). Adding labels for hypotheses within the figure could help readers quickly map hypotheses to paths.

Hypotheses

Strengths: Well structured, logically derived from the theory, and cover direct and moderating effects comprehensively.

Points for improvement: Some hypotheses are repetitive (H3a–d, H4a–d, etc.), which could be streamlined for clarity. Also, justification for expected moderation effects should be made more explicit.

Methodology

Strengths: Ethical approval is clearly stated, inclusion/exclusion criteria are appropriate, and survey procedures are transparent. Large sample size enhances generalizability.

Points for improvement: Being cross-sectional, causality is limited. Acknowledgment of this is appropriate, but considering future longitudinal design in methodology would strengthen it.

Power Analysis

Strengths: Conducted a priori with G*Power, showing rigor and planning. Achieved sample exceeds minimum requirements. Points for improvement: The rationale for assuming 10 predictors could be clarified, as could the connection between sample size and three-way exploratory interactions.

Data Collection

Strengths: Transparent recruitment process (employers + social media), clear consent procedures, anonymity ensured. Points for improvement: Phase one yielded very few responses, suggesting reliance on social media recruitment. This may introduce self-selection bias that should be discussed further.

Table 1 (Descriptive statistics)

Strengths: Provides a clear demographic overview, sample is diverse in age and professions.

Points for improvement: The predominance of women (93.4%) limits generalizability. This should be highlighted more strongly as a limitation.

Measurement Scales

Strengths: All scales are validated, translated/back-translated, and aligned with JD-R constructs. Use of OLBI is appropriate for burnout. Points for improvement: Deep acting scale may be underpowered due to only three items and two retained. This limitation is noted but should be emphasized earlier.

Statistical Analysis

Strengths: Robust use of PLS-SEM with appropriate validity checks (AVE, CR, HTMT). Clear justification for modeling emotional demands as formative. Points for improvement: The justification for choosing PLS-SEM over CB-SEM should be elaborated (beyond flexibility), especially since the study is confirmatory.

Results

Strengths: Thorough evaluation of measurement and structural models, transparent reporting of paths, effect sizes, and significance. Points for improvement: Interpretation of small effect sizes should be more cautious. Some non-significant moderation paths could be reported more briefly to reduce redundancy.

Measurement Model Evaluation (Table 2, Table 3)

Strengths: Reliability and validity tests are comprehensive, and problematic items were removed systematically. Points for improvement: The removal of items reduces comparability with prior research using the same scales. This limitation should be acknowledged more explicitly.

Structural Model & Hypothesis Testing (Figure 2, Table 4)

Strengths: Explained variance (R²) is substantial, direct effects well supported, clear reporting of moderation outcomes. Points for improvement: The unexpected positive moderating effects (meaning, support) deserve greater emphasis in both results and discussion, as they challenge assumptions of the JD-R model.

Exploratory Age Analyses

Strengths: Inclusion of linear and curvilinear age effects adds depth. Transparent reporting of null findings avoids publication bias. Points for improvement: Interpretation of the negligible U-shaped effect on disengagement could be shortened, as practical significance is minimal.

Discussion

Strengths: Links findings back to JD-R, integrates literature on “double-edged” nature of resources, and provides thoughtful explanations for failed buffering effects.

Points for improvement: The discussion is lengthy and at times repetitive. Greater focus on the most novel insights (e.g., amplification effects of resources) would enhance clarity and impact.

Implications (Theoretical & Practical)

Strengths: Theoretical contributions are clear: resources may not universally buffer demands. Practical implications highlight need for tailored interventions. Points for improvement: Practical recommendations could be expanded with more actionable strategies for organizations (e.g., training programs, systemic supports beyond individual resources).

Strengths and Limitations

Strengths: Large sample, multi-professional scope, rigorous SEM approach.

Points for improvement: Limitations should be more prominently integrated into the discussion (e.g., gender imbalance, reliance on self-reports, cross-sectional design).

Future Research

Strengths: Calls for longitudinal and multi-source data, exploration of additional resources.

Points for improvement: Could suggest integrating physiological or organizational-level indicators to overcome reliance on self-report.

Conclusions

Strengths: Accurately summarize findings, acknowledge unexpected results, and reinforce theoretical contribution. Points for improvement: Could end with a stronger forward-looking statement emphasizing the rethinking of JD-R assumptions in emotionally demanding professions.

Reviewer #2: A concise and well written paper, with some interesting conclusions.

The weaker parts of the study are:

- the participant recruitment process, which does not allow us to estimate the non-response rates and the potential bias coming from that.

- The main finding is not a breakthrough - it has been proven many times, that an emotional burden is positively associated to BO

I do not feel any revision is required.

**********

6. PLOS authors have the option to publish the peer review history of their article (what does this mean?). If published, this will include your full peer review and any attached files.

If you choose “no”, your identity will remain anonymous but your review may still be made public.

**Do you want your identity to be public for this peer review?**  For information about this choice, including consent withdrawal, please see our Privacy Policy.

Reviewer #1: **Yes:**  Carlos Santiago-Torner

Reviewer #2: No

**********

[NOTE: If reviewer comments were submitted as an attachment file, they will be attached to this email and accessible via the submission site. Please log into your account, locate the manuscript record, and check for the action link "View Attachments". If this link does not appear, there are no attachment files.]

To ensure your figures meet our technical requirements, please review our figure guidelines: https://journals.plos.org/plosone/s/figures

You may also use PLOS’s free figure tool, NAAS, to help you prepare publication quality figures: https://journals.plos.org/plosone/s/figures#loc-tools-for-figure-preparation.

NAAS will assess whether your figures meet our technical requirements by comparing each figure against our figure specifications.

# Author comment: Author response to Decision Letter 1

**DOI:** 10.1371/journal.pone.0351940.r002

22 Dec 2025

Journal Requirements

When submitting your revision, we need you to address these additional requirements.

1. Please ensure that your manuscript meets PLOS ONE's style requirements, including those for file naming. The PLOS ONE style templates can be found at https://journals.plos.org/plosone/s/file?id=wjVg/PLOSOne_formatting_sample_main_body.pdf and https://journals.plos.org/plosone/s/file?id=ba62/PLOSOne_formatting_sample_title_authors_affiliations.pdf

Response

The manuscript has been revised and reformatted to comply with PLOS ONE’s style and file-naming requirements, following the provided formatting templates. All formatting adjustments have been applied to the clean manuscript file, which has been uploaded without tracked changes.

2. We note that you have indicated that there are restrictions to data sharing for this study. For studies involving human research participant data or other sensitive data, we encourage authors to share de-identified or anonymized data. However, when data cannot be publicly shared for ethical reasons, we allow authors to make their data sets available upon request. For information on unacceptable data access restrictions, please see http://journals.plos.org/plosone/s/data-availability#loc-unacceptable-data-access-restrictions.

Before we proceed with your manuscript, please address the following prompts:

a) If there are ethical or legal restrictions on sharing a de-identified data set, please explain them in detail (e.g., data contain potentially identifying or sensitive patient information, data are owned by a third-party organization, etc.) and who has imposed them (e.g., a Research Ethics Committee or Institutional Review Board, etc.). Please also provide contact information for a data access committee, ethics committee, or other institutional body to which data requests may be sent.

b) If there are no restrictions, please upload the minimal anonymized data set necessary to replicate your study findings to a stable, public repository and provide us with the relevant URLs, DOIs, or accession numbers. Please see http://www.bmj.com/content/340/bmj.c181.long for guidelines on how to de-identify and prepare clinical data for publication. For a list of recommended repositories, please see https://journals.plos.org/plosone/s/recommended-repositories. You also have the option of uploading the data as Supporting Information files, but we would recommend depositing data directly to a data repository if possible.

Please update your Data Availability statement in the submission form accordingly.

Response

The data underlying this study cannot be made publicly available due to ethical and legal restrictions. Although the data are de-identified, they include sensitive health-related information and occupational characteristics, and public deposition would not be compliant with the approved research protocol or applicable data protection legislation.

Restrictions on public data sharing were imposed by the ethical approval granted by the Swedish Ethical Review Authority (reference number 2023-08117-01) and by the University of Gävle’s institutional data protection and research governance requirements. To ensure long-term, controlled access in line with PLOS ONE policy, the dataset has been deposited with the Swedish National Data Service (SND) under restricted access and assigned a persistent identifier (DOI: https://doi.org/10.5878/w88z-ta87).

De-identified data may be made available to qualified researchers upon reasonable request via SND, subject to institutional review and approval by the University of Gävle and, where applicable, a data access agreement. The access procedure and institutional contact information are specified in the updated Data Availability Statement in the submission system.

3. In the online submission form, you indicated that the data underlying this study contain sensitive health information and are stored in a secure repository at the University of Gävle. Due to ethical and legal restrictions, the data cannot be made publicly available. Data may be made available from the corresponding author upon reasonable request and with permission of the University of Gävle.

All PLOS journals now require all data underlying the findings described in their manuscript to be freely available to other researchers, either 1. In a public repository, 2. Within the manuscript itself, or 3. Uploaded as supplementary information.

This policy applies to all data except where public deposition would breach compliance with the protocol approved by your research ethics board. If your data cannot be made publicly available for ethical or legal reasons (e.g., public availability would compromise patient privacy), please explain your reasons on resubmission and your exemption request will be escalated for approval.

Response

We confirm that the data underlying this study cannot be made publicly available because public deposition would breach compliance with the approved research protocol and applicable data protection legislation. Although de-identified, the dataset includes sensitive health-related information and occupational characteristics, and unrestricted public access is therefore not permitted under the ethical approval granted by the Swedish Ethical Review Authority (reference number 2023-08117-01) or under the University of Gävle’s institutional data protection and research governance requirements.

Accordingly, we request an exemption from public data deposition. To ensure appropriate data availability in line with PLOS ONE policy, the dataset has been deposited with the Swedish National Data Service (SND) under restricted access and assigned a persistent identifier (DOI: https://doi.org/10.5878/w88z-ta87). De-identified data may be made available to qualified researchers via SND, subject to institutional review and approval by the University of Gävle and, where applicable, a data access agreement. The access procedure is detailed in the updated Data Availability Statement in the submission system.

4. If the reviewer comments include a recommendation to cite specific previously published works, please review and evaluate these publications to determine whether they are relevant and should be cited. There is no requirement to cite these works unless the editor has indicated otherwise.

Response

We confirm that no reviewer comments included recommendations to cite specific previously published works, and therefore no changes were made to the reference list in response to this point.

---

Response to Reviewer 1

Reviewer #1: Dear author,

I have carefully reviewed your manuscript, which addresses a highly relevant and timely issue. The paper is clearly structured and well written. At the same time, there are several areas where further refinement could enhance its clarity, theoretical contribution, and practical relevance. In what follows, I provide detailed feedback for each section with the intention of supporting the improvement of the manuscript.

Response

We would like to sincerely thank Reviewer 1 for their careful, thorough, and generous review of our manuscript. We greatly appreciate the time and effort devoted to providing such detailed and constructive feedback.

We are particularly grateful for the respectful and supportive tone of the review, as well as for the recognition of the strengths of our manuscript. The suggested revisions were clear, wise, and helpful, and they contributed to improving the paper.

We have carefully addressed all comments and suggestions. Below, we respond to each comment line by line and indicate the corresponding changes made in the manuscript.

Once again, we sincerely thank Reviewer 1 for their valuable and constructive feedback.

Point raised

Title

Strengths: Clear, informative, and well aligned with the study’s aims. It specifies the core variables and signals the moderating role of resources and age. Points for improvement: The length could be slightly reduced to increase readability. A more concise version may enhance impact, e.g., “Emotional Demands, Burnout, and the Moderating Role of Resources and Age.”

Response

Thank you for this suggestion. In response, we have revised the title to improve conciseness and readability while retaining the core constructs of the study. The title has been shortened and now reads: “Emotional demands and burnout: differential moderating effects of job resources and age.” The revised title is reflected on the title page of the manuscript.

Point raised

Abstract

Strengths: Provides a concise overview of theoretical basis (JD-R model), methods (sample, PLS-SEM), main findings, and contributions. Clear separation of direct, moderating, and exploratory effects. Points for improvement: The abstract is dense and may overwhelm readers. Simplifying some methodological details (e.g., bootstrapping, effect sizes) and emphasizing key contributions more directly would improve accessibility for a broad audience.

Response

Thank you for this constructive suggestion. In response, we have revised the abstract to reduce methodological density and improve accessibility for a broad readership. Specifically, we streamlined the description of the analytical approach, removed fine-grained methodological detail, and consolidated the presentation of results to emphasize overall patterns rather than individual effects. At the same time, we strengthened the articulation of the study’s main theoretical contribution by more clearly highlighting the limited buffering and amplifying roles of job resources, and their implications for refining key assumptions of the JD-R model. The revised abstract is provided on the second page of the manuscript.

Point raised

Keywords

Strengths: Relevant, aligned with the study’s constructs, and likely to optimize discoverability.

Points for improvement: Could benefit from including “JD-R model” explicitly, as it is central to the study.

Response

Thank you for this suggestion. We have revised the keyword list to explicitly include JD-R, which represents the central theoretical framework of the study.

Point raised

Introduction

Strengths: Comprehensive literature review, solid grounding in JD-R, clear positioning of emotional demands, and good integration of personal/organizational resources and age.

Points for improvement: The knowledge gap is somewhat implicit. It would help to explicitly highlight how this study advances beyond existing research (e.g., inconsistent findings on moderation, limited evidence for three-way interactions with age).

Response

Thank you for this helpful suggestion. In response, we revised the Introduction to make key knowledge gaps more explicit. Specifically, we added a statement highlighting the mixed and inconsistent empirical evidence regarding the buffering effects of job resources on emotional demands (p. 5). In addition, we revised the framing of the age-related discussion to emphasize the limited empirical evidence on whether such moderation effects vary across age groups (pp. 7–8).

Point raised

Figure 1 (Conceptual model)

Strengths: Presents the hypothesized relationships clearly and follows JD-R conventions.

Points for improvement: The figure should be more visually differentiated (e.g., distinct arrows for direct vs. moderating effects). Adding labels for hypotheses within the figure could help readers quickly map hypotheses to paths.

Response

Thank you for this helpful suggestion. We revised Figure 1 to improve clarity by adding hypothesis labels to all hypothesized direct and moderating paths. Age-related paths are now explicitly marked as exploratory, and these paths are visually differentiated using lighter and thinner lines.

Point raised

Hypotheses

Strengths: Well structured, logically derived from the theory, and cover direct and moderating effects comprehensively.

Points for improvement: Some hypotheses are repetitive (H3a–d, H4a–d, etc.), which could be streamlined for clarity. Also, justification for expected moderation effects should be made more explicit.

Response

Thank you for this helpful suggestion. In response, we added a brief JD–R–based rationale preceding the hypotheses to more explicitly motivate both the direct and moderating hypotheses. We also streamlined the presentation of the moderation hypotheses by grouping conceptually identical hypotheses (H3–H6) into consolidated statements while retaining the subscript notation (a–d) to preserve transparency regarding demand type and burnout dimension, thereby reducing redundancy (pp. 9-10).

Point raised

Methodology

Strengths: Ethical approval is clearly stated, inclusion/exclusion criteria are appropriate, and survey procedures are transparent. Large sample size enhances generalizability.

Points for improvement: Being cross-sectional, causality is limited. Acknowledgment of this is appropriate, but considering future longitudinal design in methodology would strengthen it.

Response

Thank you for this helpful comment. We clarified that the cross-sectional design limits both causal inference and examination of temporal processes (p. 33), and we more explicitly highlighted longitudinal and mixed-methods designs as important directions for future research (pp. 35–36).

Point raised

Power Analysis

Strengths: Conducted a priori with G*Power, showing rigor and planning. Achieved sample exceeds minimum requirements. Points for improvement: The rationale for assuming 10 predictors could be clarified, as could the connection between sample size and three-way exploratory interactions.

Response

Thank you for this helpful comment. We clarified the rationale for assuming 10 predictors and explicitly explained how the target sample size was chosen to ensure adequate power for the exploratory three-way interaction analyses (p. 11).

Point raised

Data Collection

Strengths: Transparent recruitment process (employers + social media), clear consent procedures, anonymity ensured. Points for improvement: Phase one yielded very few responses, suggesting reliance on social media recruitment. This may introduce self-selection bias that should be discussed further.

Response

Thank you for this helpful comment. We have expanded the limitations section to explicitly acknowledge the potential for self-selection bias associated with voluntary participation and the reliance on social media recruitment (p 34).

Point raised

Table 1 (Descriptive statistics)

Strengths: Provides a clear demographic overview, sample is diverse in age and professions.

Points for improvement: The predominance of women (93.4%) limits generalizability. This should be highlighted more strongly as a limitation.

Response

Thank you for this comment. We revised the limitations section to more explicitly emphasize the predominance of women in the sample as a clear limitation that restricts generalizability (p. 34).

Point raised

Measurement Scales

Strengths: All scales are validated, translated/back-translated, and aligned with JD-R constructs. Use of OLBI is appropriate for burnout. Points for improvement: Deep acting scale may be underpowered due to only three items and two retained. This limitation is noted but should be emphasized earlier.

Response

Thank you for this helpful comment. In addition to acknowledging this issue in the limitations section, we now explicitly note in the Measurement Scales section that the deep acting construct was reduced to two items following indicator evaluation, with the full rationale provided in the measurement model assessment (p. 16).

Point raised

Statistical Analysis

Strengths: Robust use of PLS-SEM with appropriate validity checks (AVE, CR, HTMT). Clear justification for modeling emotional demands as formative. Points for improvement: The justification for choosing PLS-SEM over CB-SEM should be elaborated (beyond flexibility), especially since the study is confirmatory.

Response

Thank you for this comment. We revised the justification for using PLS-SEM by explicitly contrasting it with CB-SEM and clarifying its suitability given the model structure and analytical goals (pp. 16-17).

Point raised

Results

Strengths: Thorough evaluation of measurement and structural models, transparent reporting of paths, effect sizes, and signific

Attachment Submitted filename: Response to Reviewers.docx

# Peer review: Decision Letter 1

**DOI:** 10.1371/journal.pone.0351940.r003

**Authors:** Ioana Gutu, Asim Mehmood

5 Apr 2026

PONE-D-25-45860R1

Emotional demands and burnout: differential moderating effect of job resources and age

PLOS One

Dear Dr. Vasundharaa,

Thank you for submitting your manuscript to PLOS ONE. After careful consideration, we feel that it has merit but does not fully meet PLOS ONE’s publication criteria as it currently stands. Therefore, we invite you to submit a revised version of the manuscript that addresses the points raised during the review process.

Please submit your revised manuscript by May 20 2026 11:59PM. If you will need more time than this to complete your revisions, please reply to this message or contact the journal office at plosone@plos.org. When you're ready to submit your revision, log on to https://www.editorialmanager.com/pone/ and select the 'Submissions Needing Revision' folder to locate your manuscript file.

Please include the following items when submitting your revised manuscript:

- A letter that responds to each point raised by the academic editor and reviewer(s). You should upload this letter as a separate file labeled 'Response to Reviewers'.
- A marked-up copy of your manuscript that highlights changes made to the original version. You should upload this as a separate file labeled 'Revised Manuscript with Track Changes'.
- An unmarked version of your revised paper without tracked changes. You should upload this as a separate file labeled 'Manuscript'.

If you would like to make changes to your financial disclosure, please include your updated statement in your cover letter. Guidelines for resubmitting your figure files are available below the reviewer comments at the end of this letter.

If applicable, we recommend that you deposit your laboratory protocols in protocols.io to enhance the reproducibility of your results. Protocols.io assigns your protocol its own identifier (DOI) so that it can be cited independently in the future. For instructions see: https://journals.plos.org/plosone/s/submission-guidelines#loc-laboratory-protocols. Additionally, PLOS ONE offers an option for publishing peer-reviewed Lab Protocol articles, which describe protocols hosted on protocols.io. Read more information on sharing protocols at https://plos.org/protocols?utm_medium=editorial-email&utm_source=authorletters&utm_campaign=protocols.

As the corresponding author, your ORCID iD is verified in the submission system and will appear in the published article. PLOS supports the use of ORCID, and we encourage all coauthors to register for an ORCID iD and use it as well. Please encourage your coauthors to verify their ORCID iD within the submission system before final acceptance, as unverified ORCID iDs will not appear in the published article. *Only*  the individual author can complete the verification step; PLOS staff *cannot*  verify ORCID iDs on behalf of authors.

We look forward to receiving your revised manuscript.

Kind regards,

Asim Mehmood

Academic Editor

PLOS One

Journal Requirements:

If the reviewer comments include a recommendation to cite specific previously published works, please review and evaluate these publications to determine whether they are relevant and should be cited. There is no requirement to cite these works unless the editor has indicated otherwise.

Please review your reference list to ensure that it is complete and correct. If you have cited papers that have been retracted, please include the rationale for doing so in the manuscript text, or remove these references and replace them with relevant current references. Any changes to the reference list should be mentioned in the rebuttal letter that accompanies your revised manuscript. If you need to cite a retracted article, indicate the article’s retracted status in the References list and also include a citation and full reference for the retraction notice.

[Note: HTML markup is below. Please do not edit.]

Reviewers' comments:

Reviewer's Responses to Questions

**Comments to the Author**

1. If the authors have adequately addressed your comments raised in a previous round of review and you feel that this manuscript is now acceptable for publication, you may indicate that here to bypass the “Comments to the Author” section, enter your conflict of interest statement in the “Confidential to Editor” section, and submit your "Accept" recommendation.

Reviewer #2: All comments have been addressed

Reviewer #3: (No Response)

**********

2. Is the manuscript technically sound, and do the data support the conclusions?

The manuscript must describe a technically sound piece of scientific research with data that supports the conclusions. Experiments must have been conducted rigorously, with appropriate controls, replication, and sample sizes. The conclusions must be drawn appropriately based on the data presented.

Reviewer #2: Yes

Reviewer #3: Yes

**********

3. Has the statistical analysis been performed appropriately and rigorously?

Reviewer #2: Yes

Reviewer #3: Yes

**********

4. Have the authors made all data underlying the findings in their manuscript fully available?

The PLOS Data policy requires authors to make all data underlying the findings described in their manuscript fully available without restriction, with rare exception (please refer to the Data Availability Statement in the manuscript PDF file). The data should be provided as part of the manuscript or its supporting information, or deposited to a public repository. For example, in addition to summary statistics, the data points behind means, medians and variance measures should be available. If there are restrictions on publicly sharing data—e.g. participant privacy or use of data from a third party—those must be specified.

Reviewer #2: Yes

Reviewer #3: Yes

**********

5. Is the manuscript presented in an intelligible fashion and written in standard English?

PLOS ONE does not copyedit accepted manuscripts, so the language in submitted articles must be clear, correct, and unambiguous. Any typographical or grammatical errors should be corrected at revision, so please note any specific errors here.

Reviewer #2: Yes

Reviewer #3: Yes

**********

6. Review Comments to the Author

Please use the space provided to explain your answers to the questions above. You may also include additional comments for the author, including concerns about dual publication, research ethics, or publication ethics. (Please upload your review as an attachment if it exceeds 20,000 characters)

Reviewer #2: I am satisfied with the author's time and effort to produce the initial as well as the revised version of their paper.

Reviewer #3: Dear authors,

At the outset, I congratulate you for this initiative. In your modified manuscript which has most of the corrections from first reviewer, I have following comments :

- Please share the EQUATOR guidelines to report the rigor of this study. this could be attached as a supplement as well.

- it would be a great asset to state that how the findings of the study could be help bridge the gap of the study which could come out strongly in the manuscript as well and probably would benefit from a figure too.

- Is there something as a strong outcome that could be implemented coming out from the study. This could be reflected well in discussion section.

All the best!

**********

7. PLOS authors have the option to publish the peer review history of their article (what does this mean?). If published, this will include your full peer review and any attached files.

If you choose “no”, your identity will remain anonymous but your review may still be made public.

**Do you want your identity to be public for this peer review?**  For information about this choice, including consent withdrawal, please see our Privacy Policy.

Reviewer #2: No

Reviewer #3: **Yes:**  Vasundharaa S Nair

**********

[NOTE: If reviewer comments were submitted as an attachment file, they will be attached to this email and accessible via the submission site. Please log into your account, locate the manuscript record, and check for the action link "View Attachments". If this link does not appear, there are no attachment files.]

To ensure your figures meet our technical requirements, please review our figure guidelines: https://journals.plos.org/plosone/s/figures

You may also use PLOS’s free figure tool, NAAS, to help you prepare publication quality figures: https://journals.plos.org/plosone/s/figures#loc-tools-for-figure-preparation.

NAAS will assess whether your figures meet our technical requirements by comparing each figure against our figure specifications.

# Author comment: Author response to Decision Letter 2

**DOI:** 10.1371/journal.pone.0351940.r004

8 Apr 2026

Dear Dr. Mehmood,

We are pleased to resubmit our revised manuscript entitled “Emotional demands and burnout: differential moderating effect of job resources and age” (Manuscript ID: PONE-D-25-45860R1) for consideration in PLOS ONE.

We would like to thank you and the reviewers for your constructive and insightful comments. We have carefully revised the manuscript in response to all points raised. In particular, we have (1) enhanced the theoretical contribution by clarifying how the findings address a gap in the Job Demands–Resources (JD–R) literature, (2) expanded the discussion of practical implications, and (3) improved reporting transparency by adhering to the STROBE guidelines and including the completed checklist as supplementary material.

We believe that these revisions have strengthened the clarity, rigor, and contribution of the manuscript. A detailed, point-by-point response to the reviewers’ comments is provided in the accompanying “Response to Reviewers” document, and all changes are highlighted in the tracked version of the manuscript.

We hope that the revised manuscript is now suitable for publication in PLOS ONE, and we look forward to your decision.

Kind regards,

Tímea Zsuzsanna Popucza

--

Reviewer #2

We thank the reviewer for their positive evaluation of the manuscript and for acknowledging the revisions made. We are pleased that the reviewer is satisfied with the current version.

Reviewer #3

We thank the reviewer for their thoughtful and constructive comments, as well as for the positive feedback on the revisions made in the previous round. We appreciate the opportunity to further strengthen the manuscript and have addressed each of the points raised below.

Comment:

“- Please share the EQUATOR guidelines to report the rigor of this study. this could be attached as a supplement as well.”

Response:

Thank you for this valuable suggestion. To enhance transparency and reporting rigor, we have ensured that the manuscript adheres to the STROBE guidelines for cross-sectional studies. A completed STROBE checklist has been added as Supplementary Material (S1 File), indicating where each reporting item is addressed in the manuscript. In addition, a statement has been included in the Methods section (lines 222-224) to clarify that the study is reported in accordance with these guidelines.

Comment:

“-it would be a great asset to state that how the findings of the study could be help bridge the gap of the study which could come out strongly in the manuscript as well and probably would benefit from a figure too.”

Response:

Thank you for this insightful suggestion. To make the theoretical contribution more explicit, we have revised the opening of the Discussion section (lines 515–522) to more clearly articulate how the findings address a gap in the Job Demands–Resources (JD–R) literature, particularly by showing that job resources may not uniformly buffer the effects of emotional demands and may, in some cases, be associated with amplification effects. To avoid redundancy with the existing figures, we addressed this point through a strengthened conceptual explanation in the text rather than adding an additional figure.

Comment:

- Is there something as a strong outcome that could be implemented coming out from the study. This could be reflected well in discussion section.

Response:

Thank you for this helpful suggestion. To more clearly articulate the practical implications, we have expanded the Implications section (lines 737–746) to highlight that interventions in emotionally demanding professions may need to go beyond strengthening job resources alone and consider how emotional demands themselves are structured and managed. This addition clarifies how the findings can inform practice while maintaining an appropriately cautious interpretation.

--

We sincerely thank the Academic Editor and both reviewers for their valuable feedback, which has helped improve the clarity and contribution of the manuscript.

Attachment Submitted filename: Response to Reviewers..docx

# Peer review: Decision Letter 2

**DOI:** 10.1371/journal.pone.0351940.r005

**Authors:** Ioana Gutu, Asim Mehmood, Bo Hu

1 Jun 2026

PONE-D-25-45860R2

Emotional demands and burnout: differential moderating effect of job resources and age

PLOS One

Dear Dr. Popucza,

Thank you for submitting your manuscript to PLOS ONE. After careful consideration, we feel that it has merit but does not fully meet PLOS ONE’s publication criteria as it currently stands. Therefore, we invite you to submit a revised version of the manuscript that addresses the points raised during the review process.

Please submit your revised manuscript by Jul 16 2026 11:59PM. If you will need more time than this to complete your revisions, please reply to this message or contact the journal office at plosone@plos.org. When you're ready to submit your revision, log on to https://www.editorialmanager.com/pone/ and select the 'Submissions Needing Revision' folder to locate your manuscript file.

Please include the following items when submitting your revised manuscript:

- A letter that responds to each point raised by the academic editor and reviewer(s). You should upload this letter as a separate file labeled 'Response to Reviewers'.
- A marked-up copy of your manuscript that highlights changes made to the original version. You should upload this as a separate file labeled 'Revised Manuscript with Track Changes'.
- An unmarked version of your revised paper without tracked changes. You should upload this as a separate file labeled 'Manuscript'.

If you would like to make changes to your financial disclosure, please include your updated statement in your cover letter. Guidelines for resubmitting your figure files are available below the reviewer comments at the end of this letter.

If applicable, we recommend that you deposit your laboratory protocols in protocols.io to enhance the reproducibility of your results. Protocols.io assigns your protocol its own identifier (DOI) so that it can be cited independently in the future. For instructions see: https://journals.plos.org/plosone/s/submission-guidelines#loc-laboratory-protocols. Additionally, PLOS ONE offers an option for publishing peer-reviewed Lab Protocol articles, which describe protocols hosted on protocols.io. Read more information on sharing protocols at https://plos.org/protocols?utm_medium=editorial-email&utm_source=authorletters&utm_campaign=protocols.

As the corresponding author, your ORCID iD is verified in the submission system and will appear in the published article. PLOS supports the use of ORCID, and we encourage all coauthors to register for an ORCID iD and use it as well. Please encourage your coauthors to verify their ORCID iD within the submission system before final acceptance, as unverified ORCID iDs will not appear in the published article. *Only*  the individual author can complete the verification step; PLOS staff *cannot*  verify ORCID iDs on behalf of authors.

We look forward to receiving your revised manuscript.

Kind regards,

Bo Hu

Academic Editor

PLOS One

Journal Requirements:

If the reviewer comments include a recommendation to cite specific previously published works, please review and evaluate these publications to determine whether they are relevant and should be cited. There is no requirement to cite these works unless the editor has indicated otherwise.

Please review your reference list to ensure that it is complete and correct. If you have cited papers that have been retracted, please include the rationale for doing so in the manuscript text, or remove these references and replace them with relevant current references. Any changes to the reference list should be mentioned in the rebuttal letter that accompanies your revised manuscript. If you need to cite a retracted article, indicate the article’s retracted status in the References list and also include a citation and full reference for the retraction notice.

Additional Editor Comments :

The reviewers are largely satisfied with the revised manuscript and have indicated that their major concerns have been adequately addressed. I also consider the manuscript to be substantially improved and close to being suitable for publication.

Before a final decision can be made, however, I would like to request a small number of additional editorial revisions. These points are intended to improve reporting clarity, consistency, and readiness for publication.

Values currently reported as p = 0.000 should be changed to p < .001 or p < 0.001, as appropriate. Please ensure that this formatting is applied consistently in the text, tables, and figures.

Please check the formatting of statistical notation throughout the manuscript. For example, R² = 0,575 and adjusted R² = 0,571. These should be revised to decimal points, for example R² = 0.575 and adjusted R² = 0.571.

Please carefully verify the values reported for Deep Acting in Table 2. The reported loading range for Deep Acting appears difficult to reconcile with the reported AVE and composite reliability values. Please check whether the final retained item loadings, AVE, Cronbach’s alpha, ρa, and ρc are all taken from the same final measurement model.

In the paragraph discussing the measurement model, please correct the cross-reference if needed. The text appears to refer to “Table 1” when the construct-level metrics are actually presented in Table 2.

The text currently states that all constructs showed Cronbach’s alpha and composite reliability values above .70, but Deep Acting is reported with Cronbach’s alpha = .691.

The STROBE checklist notes that missing data are described but not reported per variable. Please either report the number of missing values for each variable.

Please carefully proofread the Acknowledgments and final sections for minor typographical and punctuation errors. For example, there appear to be duplicated or incorrectly punctuated phrases such as “University of Gävle.Support,” “professions. .,” and “over time..”. These should be corrected before publication.

Please ensure that the final figure files and figure captions are consistent with the revised manuscript.

[Note: HTML markup is below. Please do not edit.]

Reviewers' comments:

Reviewer's Responses to Questions

**Comments to the Author**

1. If the authors have adequately addressed your comments raised in a previous round of review and you feel that this manuscript is now acceptable for publication, you may indicate that here to bypass the “Comments to the Author” section, enter your conflict of interest statement in the “Confidential to Editor” section, and submit your "Accept" recommendation.

Reviewer #2: All comments have been addressed

**********

2. Is the manuscript technically sound, and do the data support the conclusions?

The manuscript must describe a technically sound piece of scientific research with data that supports the conclusions. Experiments must have been conducted rigorously, with appropriate controls, replication, and sample sizes. The conclusions must be drawn appropriately based on the data presented.

Reviewer #2: Yes

**********

3. Has the statistical analysis been performed appropriately and rigorously?

Reviewer #2: Yes

**********

4. Have the authors made all data underlying the findings in their manuscript fully available?

The PLOS Data policy requires authors to make all data underlying the findings described in their manuscript fully available without restriction, with rare exception (please refer to the Data Availability Statement in the manuscript PDF file). The data should be provided as part of the manuscript or its supporting information, or deposited to a public repository. For example, in addition to summary statistics, the data points behind means, medians and variance measures should be available. If there are restrictions on publicly sharing data—e.g. participant privacy or use of data from a third party—those must be specified.

Reviewer #2: Yes

**********

5. Is the manuscript presented in an intelligible fashion and written in standard English?

PLOS ONE does not copyedit accepted manuscripts, so the language in submitted articles must be clear, correct, and unambiguous. Any typographical or grammatical errors should be corrected at revision, so please note any specific errors here.

Reviewer #2: Yes

**********

6. Review Comments to the Author

Please use the space provided to explain your answers to the questions above. You may also include additional comments for the author, including concerns about dual publication, research ethics, or publication ethics. (Please upload your review as an attachment if it exceeds 20,000 characters)

Reviewer #2: I shall stand by my previous review. As tempting as it may be to become a co-author of this paper, I feel that the author has made a sufficient effort for their work to be accepted for publication.

**********

7. PLOS authors have the option to publish the peer review history of their article (what does this mean?). If published, this will include your full peer review and any attached files.

If you choose “no”, your identity will remain anonymous but your review may still be made public.

**Do you want your identity to be public for this peer review?**  For information about this choice, including consent withdrawal, please see our Privacy Policy.

Reviewer #2: **Yes:**  Mr Paweł Borysiewicz

**********

[NOTE: If reviewer comments were submitted as an attachment file, they will be attached to this email and accessible via the submission site. Please log into your account, locate the manuscript record, and check for the action link "View Attachments". If this link does not appear, there are no attachment files.]

To ensure your figures meet our technical requirements, please review our figure guidelines: https://journals.plos.org/plosone/s/figures

You may also use PLOS’s free figure tool, NAAS, to help you prepare publication quality figures: https://journals.plos.org/plosone/s/figures#loc-tools-for-figure-preparation.

NAAS will assess whether your figures meet our technical requirements by comparing each figure against our figure specifications.

# Author comment: Author response to Decision Letter 3

**DOI:** 10.1371/journal.pone.0351940.r006

2 Jun 2026

Dear Dr. Hu,

We would like to thank you for your careful evaluation of our manuscript, Emotional demands and burnout: differential moderating effect of job resources and age [PONE-D-25-45860R2] - [EMID:1a52517e7f43393a], and for your constructive comments. We appreciate your positive assessment of the revisions made thus far and are grateful for the opportunity to further improve the manuscript. We would also like to thank Reviewer #2 for the careful evaluation of the manuscript and for the constructive feedback provided throughout the review process. We appreciate the reviewer’s time and effort in helping to improve the quality and clarity of this work.

In response to the additional editorial comments, we have carefully revised the manuscript and addressed each point raised. The changes made are detailed below.

Values currently reported as p = 0.000 should be changed to p < .001 or p < 0.001, as appropriate. Please ensure that this formatting is applied consistently in the text, tables, and figures.

Response:

Thank you for this observation. We have revised the reporting of p-values throughout the manuscript and replaced all instances of p = 0.000 with p < .001, as appropriate. These changes have been applied consistently in the text, tables, and figures.

Please check the formatting of statistical notation throughout the manuscript. For example, R² = 0,575 and adjusted R² = 0,571. These should be revised to decimal points, for example R² = 0.575 and adjusted R² = 0.571.

Response:

Thank you for this observation. We have reviewed the statistical notation throughout the manuscript and corrected all instances where decimal commas were used instead of decimal points. These corrections have been applied consistently in the text, tables, and figures, including Figure 2.

Please carefully verify the values reported for Deep Acting in Table 2. The reported loading range for Deep Acting appears difficult to reconcile with the reported AVE and composite reliability values. Please check whether the final retained item loadings, AVE, Cronbach’s alpha, ρa, and ρc are all taken from the same final measurement model.

Response:

Thank you for identifying this inconsistency. Upon re-examining the measurement model output, we found that the loading ranges reported in Table 2 corresponded to the initial item loadings rather than the final loadings obtained after model estimation. The AVE, Cronbach’s alpha, ρa, and composite reliability (ρc) values were correctly reported from the final measurement model. We have therefore updated the loading ranges for all constructs in Table 2 so that all reported metrics are derived consistently from the same final measurement model. This correction does not affect the results or conclusions of the study.

In the paragraph discussing the measurement model, please correct the cross-reference if needed. The text appears to refer to “Table 1” when the construct-level metrics are actually presented in Table 2.

Response:

Thank you for identifying this error. We have corrected the cross-reference in the manuscript. The paragraph discussing the measurement model now correctly refers to Table 2.

The text currently states that all constructs showed Cronbach’s alpha and composite reliability values above .70, but Deep Acting is reported with Cronbach’s alpha = .691.

Response:

Thank you for identifying this inconsistency. We have revised the text describing the reliability results to reflect the reported values accurately. The revised wording acknowledges that Deep Acting showed a Cronbach’s alpha slightly below the conventional threshold, while its composite reliability and average variance extracted remained above recommended levels.

The STROBE checklist notes that missing data are described but not reported per variable. Please either report the number of missing values for each variable.

Response:

Thank you for this observation. We have revised the Methods section to report the extent of missing data for the variables included in the analyses. Missing data were minimal, with one missing response for Disengagement 3, five missing responses for Deep Acting 2, and one missing response for Deep Acting 1. We also updated the STROBE checklist accordingly.

Please carefully proofread the Acknowledgments and final sections for minor typographical and punctuation errors. For example, there appear to be duplicated or incorrectly punctuated phrases such as “University of Gävle.Support,” “professions. .,” and “over time..”. These should be corrected before publication.

Response:

Thank you for identifying these issues. We carefully proofread the Acknowledgments and final sections of the manuscript and corrected typographical, spacing, and punctuation errors, including duplicated punctuation marks and formatting inconsistencies where present.

Please ensure that the final figure files and figure captions are consistent with the revised manuscript.

Response:

Thank you for this reminder. We reviewed all figure files and figure captions and verified that they are consistent with the revised manuscript. Where necessary, statistical notations were updated to ensure consistency with the text and tables. The final figures have been provided in EPS format in accordance with the journal’s publication requirements and in PNG format for viewing purposes.

Attachment Submitted filename: Response to Reviewer_R3.docx

# Editor report: Decision Letter 3

**DOI:** 10.1371/journal.pone.0351940.r007

**Authors:** Ioana Gutu, Asim Mehmood, Bo Hu, Bo Hu

3 Jun 2026

<p>Emotional demands and burnout: differential moderating effect of job resources and age

PONE-D-25-45860R3

Dear Dr. Popucza,

We’re pleased to inform you that your manuscript has been judged scientifically suitable for publication and will be formally accepted for publication once it meets all outstanding technical requirements.

Within one week, you’ll receive an e-mail detailing the required amendments. When these have been addressed, you’ll receive a formal acceptance letter and your manuscript will be scheduled for publication.

An invoice will be generated when your article is formally accepted. Please note, if your institution has a publishing partnership with PLOS and your article meets the relevant criteria, all or part of your publication costs will be covered. Please make sure your user information is up-to-date by logging into Editorial Manager at Editorial Manager® and clicking the ‘Update My Information' link at the top of the page. For questions related to billing, please contact billing support.

If your institution or institutions have a press office, please notify them about your upcoming paper to help maximize its impact. If they’ll be preparing press materials, please inform our press team as soon as possible -- no later than 48 hours after receiving the formal acceptance. Your manuscript will remain under strict press embargo until 2 pm Eastern Time on the date of publication. For more information, please contact onepress@plos.org.

Kind regards,

Bo Hu

Academic Editor

PLOS One

Additional Editor Comments (optional):

Reviewers' comments:

# Editor report: Acceptance letter

**DOI:** 10.1371/journal.pone.0351940.r008

**Authors:** Ioana Gutu, Asim Mehmood, Bo Hu, Bo Hu

PONE-D-25-45860R3

PLOS One

Dear Dr. Popucza,

I'm pleased to inform you that your manuscript has been deemed suitable for publication in PLOS One. Congratulations! Your manuscript is now being handed over to our production team.

At this stage, our production department will prepare your paper for publication. This includes ensuring the following:

* All references, tables, and figures are properly cited

* All relevant supporting information is included in the manuscript submission,

* There are no issues that prevent the paper from being properly typeset

You will receive further instructions from the production team, including instructions on how to review your proof when it is ready. Please keep in mind that we are working through a large volume of accepted articles, so please give us a few days to review your paper and let you know the next and final steps.

Lastly, if your institution or institutions have a press office, please let them know about your upcoming paper now to help maximize its impact. If they'll be preparing press materials, please inform our press team within the next 48 hours. Your manuscript will remain under strict press embargo until 2 pm Eastern Time on the date of publication. For more information, please contact onepress@plos.org.

You will receive an invoice from PLOS for your publication fee after your manuscript has reached the completed accept phase. If you receive an email requesting payment before acceptance or for any other service, this may be a phishing scheme. Learn how to identify phishing emails and protect your accounts at https://explore.plos.org/phishing.

If we can help with anything else, please email us at customercare@plos.org.

Thank you for submitting your work to PLOS ONE and supporting open access.

Kind regards,

PLOS ONE Editorial Office Staff

on behalf of

Dr. Bo Hu

Academic Editor

PLOS One
